# Multimodal Artificial Intelligence for Precision Critical Care: A Scoping Review

**DOI:** 10.34133/hds.0356

**Published:** 2026-04-16

**Authors:** Meicheng Yang, Nan Shi, Hui Chen, Erica Yan Ping Po, Pauline Yeung Ng, Peter Chi Keung Lai, Yik-Chung Wu, Daniel Yee Tak Fong

**Affiliations:** ^1^ School of Nursing, Li Ka Shing Faculty of Medicine, The University of Hong Kong, Hong Kong Special Administrative Region, China.; ^2^ Jiangsu Provincial Key Laboratory of Critical Care Medicine, Department of Critical Care Medicine, Zhongda Hospital, School of Medicine, Southeast University, Nanjing, China.; ^3^ Critical Care Medicine Unit, School of Clinical Medicine, The University of Hong Kong, Hong Kong Special Administrative Region, China.; ^4^ Department of Adult Intensive Care, Queen Mary Hospital, Hong Kong Special Administrative Region, China.; ^5^ Department of Electrical and Electronic Engineering, The University of Hong Kong, Hong Kong Special Administrative Region, China.

## Abstract

**Background:** Intensive care units (ICUs) generate large, heterogeneous data from critically ill patients. Multimodal artificial intelligence (AI), which integrates diverse data modalities within unified models, offers substantial potential for precision medicine in critical care. However, comprehensive reviews of its current progress, methods, and challenges remain scarce. **Methods:** This scoping review systematically surveys the literature on multimodal AI models in critical care, following the Preferred Reporting Items for Systematic reviews and Meta-Analyses extension for Scoping Reviews guidelines. A search was conducted across the PubMed, EMBASE, Scopus, Web of Science, and IEEE Xplore databases between 2010 and 2025. We included studies that reported integrating at least 2 data modalities using AI to evaluate outcomes or tasks relevant to critical care. **Results:** We included 86 studies, 85% published within the last 5 years. Five data modalities were reported, most commonly structured data (*n* = 80) and text (*n* = 54), followed by imaging (28), waveforms (16), and videos (2). Intermediate fusion was the predominant strategy for integrating multiple modalities, used in 54 studies. We identified 63 feature extraction methods before modality fusion, 21 modeling approaches applied to 35 distinct clinical outcome tasks after fusion, and 14 explainability techniques. Thirty-four studies shared code, and 9 open-source multimodal ICU databases were identified. Overall, multimodal AI outperformed unimodal models, achieving a 4.4% relative improvement in the area under the curve for diagnosis or prognosis prediction tasks. **Conclusion:** Drawing on the latest evidence, this review provides a strategic roadmap for the design, evaluation, and implementation of multimodal AI systems in the ICU, with the ultimate aim of improving patient outcomes.

## Introduction

Intensive care units (ICUs) are specialized hospital units dedicated to delivering highly coordinated care to patients who have, or are at risk of, life-threatening organ dysfunction [1]. Patients admitted to ICUs have a high risk of in-hospital mortality and require substantial medical resources. In the United States, over 5 million patients are admitted to ICUs annually, with adult ICU mortality rates average ranging from 10% to 29%, depending on age, comorbidities, and severity of illness [2]. The management of these critically ill patients requires advanced medical and nursing support, continuous physiologic monitoring, and a variety of organ support therapies [3]. Accordingly, modern ICUs generate large and diverse amounts of patient data during the care processes.

Electronic health records (EHRs) include both structured data, including vital signs, laboratory results, medications, and clinical interventions, and unstructured data from free-text clinical notes. Continuous bedside monitors provide a range of physiological data such as electrocardiograms (ECGs), respiratory waveforms, arterial blood pressure (ABP) waveforms, and electroencephalograms (EEGs) for neurological monitoring. In addition, medical imaging such as x-rays, computed tomography (CT), or magnetic resonance imaging (MRI) generates visual outputs for diagnosis. Moreover, research settings are increasingly incorporating omics data to help define subtypes of critical illness [4–6], further enriching the clinical information available for patient assessment and decision-making. This large volume of heterogeneous data offers substantial potential for advancing precision medicine to inform clinical decision-making in the ICU, yet it exceeds the ability of clinicians to process and interpret all available information in real time [7,8]. Therefore, the development of advanced computational methods is essential to effectively integrate multimodal data sources and extract clinically meaningful insights.

Most current applications of artificial intelligence (AI) in critical care focus on addressing specific clinical tasks using a single data modality, such as using structured EHR data for sepsis prediction [9], patient subphenotyping [10,11], or medication dosing optimization [12,13], and utilizing medical imaging, such as MRI for neurological outcome assessment [14,15]. However, these single-modality models fail to capture the full complexity of critical illness, as they do not reflect the way clinicians integrate information from multiple sources and modalities when making diagnoses, assessing prognoses, and formulating treatment plans [16]. Multimodal AI refers to algorithms that can simultaneously process, integrate, and analyze 2 or more data modalities within a unified model. By using various fusion strategies designed to integrate information from multiple data modalities effectively, multimodal models allow for the compensation of information that may be missing or incomplete in any single modality, thus providing more comprehensive and clinically relevant insights into a patient’s condition that can match the cognitive behavior of healthcare providers [7,8,16,17]. An increasing number of studies are exploring the approach of multimodal AI models in critical care. For example, Xu et al. [18] demonstrated the feasibility of predicting mortality and ICU length of stay by integrating clinical variables and ECG waveforms within an AI model. Amiri et al. [19] combined functional MRI and EEG data to predict residual consciousness, while Khader et al. [20] utilized chest x-rays together with 15 clinical variables to develop a model for predicting pathological conditions. Lin et al. [21] simultaneously incorporated clinical risk factors, chest x-rays, and radiology report text data to predict ICU mortality. All of these studies have shown that multimodal models outperform single-modality models in predictive performance. Given that multimodal AI-based models in critical care are rapidly evolving, a comprehensive systematic review of the relevant literature is urgently needed.

Some previous reviews have provided broad overviews of multimodal AI in medicine or healthcare [7,8,16,17,22], and others have focused specifically on the integration of biomedical images and clinical EHR data [23–25] or domains such as oncology [26,27] and cancer biomarker discovery [28]. However, to the best of our knowledge, no review has systematically summarized the methodologies and processes specific to the application of multimodal AI in critical care.

Therefore, this scoping review aims to comprehensively map the current landscape of multimodal AI for precision critical care. We summarize recent developments by systematically examining the types of data modality and AI methods utilized, the clinical problems addressed, the availability of public databases, and strategies for explainability. By consolidating the state of the art, this review seeks to highlight both the progress made and the remaining critical gaps, while also outlining barriers to clinical implementation and providing recommendations for future research and practice. Ultimately, our goal is to facilitate the advancement of multimodal, data-driven precision medicine in critical care.

## Methods

### Methodological framework and research questions

This scoping review was conducted in accordance with the methodological framework proposed by Arksey and O’Malley [29], further refined by Levac et al. [30], and Peters et al. [31]. The reporting of this review follows the Preferred Reporting Items for Systematic reviews and Meta-Analyses extension for Scoping Reviews checklist [32]. The protocol for this review was prospectively registered on the Open Science Framework [33].

This review aimed to address the following key questions: First, what types of data modalities and their combinations, as well as fusion methods, analysis techniques, and machine learning models, have been applied in multimodal AI studies conducted within critical care settings? Second, what are the current clinical applications of multimodal AI in critical care, and to what extent do they improve patient outcomes and demonstrate diagnostic accuracy? Third, what implementation challenges and gaps exist in deploying multimodal AI in critical care, and what future directions should be pursued to address these limitations?

### Information sources and search strategy

A comprehensive literature search was performed on 2025 May 4 using multiple databases: PubMed, EMBASE, Scopus, Web of Science, and IEEE Xplore. Searches were restricted to articles published between 2010 January and 2025 April to capture recent methodological advances. Searches were performed using subject headings, keywords, and related synonyms from a combination of the following terms: critical care (“critical care”, “intensive care”, “ICU”, “critical illness”, “critically ill”, and “acute care”) with terms for machine learning (“machine learning”, “deep learning”, “artificial intelligence”, “AI”, and “neural network”), and terms indicating multimodal data integration (“multimodal”, “multi-modal”, “multiple modalities”, “multiple data”, “multi-view”, “multi-source”, “cross-modal”, and “data fusion”). The full search strategy for each database is provided in Table A1. In addition, reference lists of all included studies and pertinent review articles were manually screened to identify further eligible publications.

**Fig. 1.**
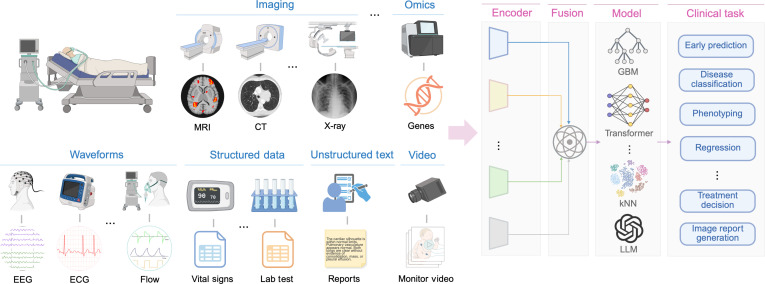
Conceptual overview of multimodal data collection and machine learning workflow in critical care. CT, computed tomography; EEG, electroencephalogram; ECG, electrocardiogram; GBM, gradient boosting machine; kNN = *k*-nearest neighbors; MRI, magnetic resonance imaging; LLM, large language model.

We used broad operational definitions for each data modality within the multimodal framework based on the type of data representation. Data modalities relevant to ICU settings could be categorized into medical imaging (e.g., CT, MRI, and x-ray), waveforms (e.g., ECG, EEG, ABP, and audio), structured data (e.g., demographics, vital signs, and laboratory results), unstructured text (e.g., clinical notes and medical image reports), omics (e.g., genomics, transcriptomics and proteomics), and video data (e.g., continuous record of frames from camera) (Fig. 1).

### Study selection

Citations retrieved from the 5 database searches were imported into Covidence, a reference management platform, where duplicates were identified and removed.

Studies were eligible for inclusion if they included patients admitted to the ICU, applied machine learning or deep learning techniques, integrated at least 2 distinct data modalities, and evaluated outcomes or tasks relevant to critical care. We excluded studies that were conference abstracts lacking sufficient methodological details, review articles, non-English publications, studies based on nonhuman data, investigations primarily focused on system or platform development unrelated to our topic, and preprints not published in peer-reviewed journals or conference proceedings.

Two reviewers (M.Y. and N.S.) screened the titles and abstracts of all retrieved records independently to assess their eligibility based on predefined inclusion criteria. Subsequently, full-text articles of potentially relevant studies were then independently evaluated by the same reviewers. Any disagreements between the reviewers at any stage of the selection process were resolved through discussion or consultation with a third reviewer (H.C.).

### Data extraction

We developed a standardized data extraction form to systematically capture relevant information from each eligible study. For each included study, 2 independent reviewers (M.Y. and N.S.) extracted key information, including the first author, year of publication, country of the first author’s institution, data sources, target patient population, sample size, study design, and any guidelines followed in model development. Regarding data modalities and modeling approaches, we collected details on the types of data modalities utilized, fusion strategies used, models applied to individual modalities prior to fusion, models used for or after data fusion, methods for addressing missing modalities or missing values within each modality, and methods for addressing class imbalance for diagnosis or prognosis prediction. For model validation and clinical application, we extracted information on target clinical outcomes, validation procedures, comparative performance between multimodal and unimodal approaches, the use of explainability techniques, whether model performance was compared with that of clinicians, and the availability of open-source code.

**Fig. 2.**
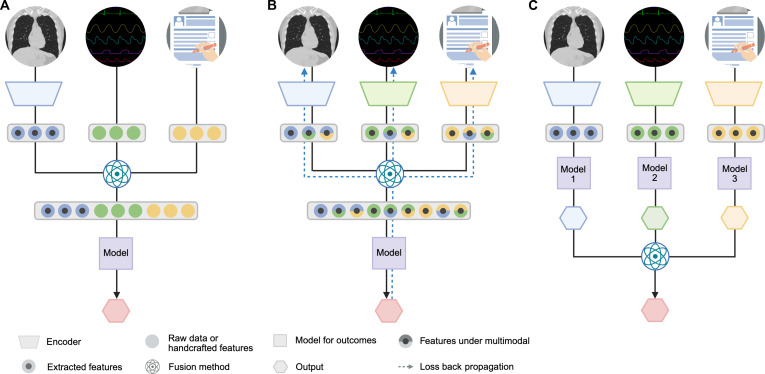
Multimodal data fusion strategies. (A) Early fusion, most features are raw data or handcrafted but may also be deep features extracted by frozen encoders (i.e., features not updated by loss back-propagation). (B) Intermediate fusion. (C) Late fusion.

We classified fusion strategies into the following 4 categories [7,23,27]: first, early fusion, which integrates information from all modalities at the input level and feeds it into a single model (Fig. 2A); second, intermediate fusion, which constructs modality-specific representations and models intermodal interactions prior to joint learning, with the loss propagated back during training to iteratively update all feature representations (Fig. 2B); third, late fusion, where separate models are trained for each modality and their predictions are combined to generate the final output (Fig. 2C); and fourth, mixed fusion refers to the combination of 2 or more of the above fusion strategies (Fig. A1) [7].

### Data analysis and presentation

Given the heterogeneity across studies in terms of methodologies, data modalities, fusion strategies, and clinical tasks, a formal meta-analysis was deemed inappropriate. Instead, we performed a narrative synthesis and descriptive summary of the findings, aligning with standard practice for scoping reviews. The results were systematically categorized and presented according to thematic groupings, specifically by data modality combinations, AI models (fusion strategies and algorithms used before and after fusion), targeted populations, explainable methods, and clinical outcomes. For studies using multiple fusion strategies or AI models, we reported the approach that achieved the best performance.

The findings were depicted in figures and summarized in structured tables to aid interpretation and improve readability. To compare each study’s final multimodal model with its unimodal baselines, the unimodal benchmark was selected as the best-performing single-modality model, and was mainly quantified with the area under the curve (AUC) and expressed as the relative gain: 100 × (AUC_multimodal_ − AUC_unimodal_) / AUC_unimodal_. When a study reported AUC values for more than 2 clinical tasks, we calculated the mean across tasks to obtain a composite estimate. We then report the mean relative increase in AUC and the associated 95% confidence intervals (CIs) across all studies.

## Results

### Study selection and characteristics

**Fig. 3.**
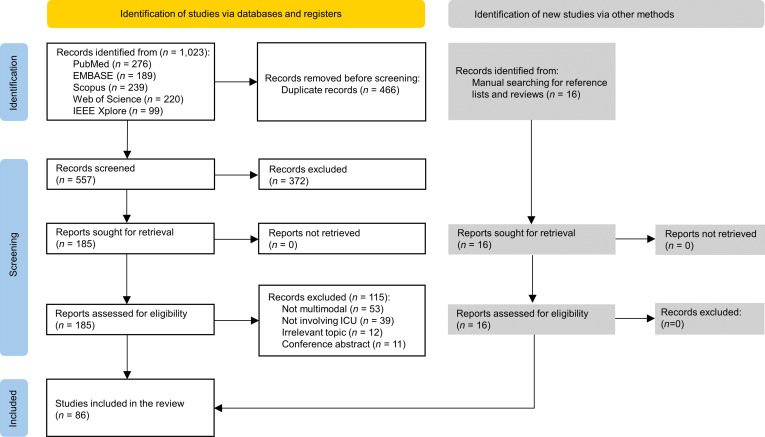
Flowchart summarizing the study selection process.

A comprehensive search across 5 databases was completed in 2025 May, identifying 1,023 records (Fig. 3). After the removal of 466 duplicates by Covidence, 185 articles were retained following title and abstract screening. After the full-text screening, 70 studies met the eligibility criteria. An additional 16 studies were identified through manual screening of reference lists from included articles and relevant reviews. Finally, 86 studies fulfilled our inclusion criteria and were included in the scoping review (Table A2).

**Fig. 4.**
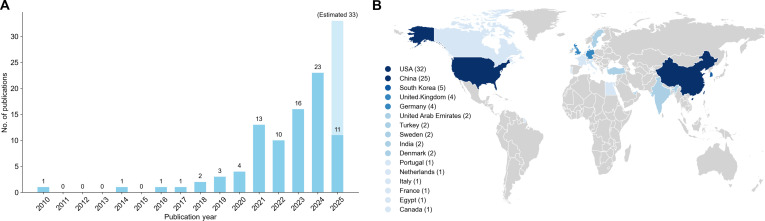
Study characteristics. (A) Temporal distribution of published studies from 2010 to 2025 April. (B) Geographic graph of country of first-author affiliations.

Among the 86 included studies, 73 (85%) studies were published within the past 5 years, underscoring the burgeoning interest in multimodal AI for critical care (Fig. 4A). In addition, first-author affiliations were most frequently based in the United States (*n* = 32), followed by China (*n* = 25). The remaining 14 countries collectively accounted for 29 studies, with South Korea leading this group (*n* = 5) (Fig. 4B). A total of 79 studies used a retrospective study design, 3 were developed retrospectively and subsequently validated in prospective cohorts, and 4 were conducted prospectively. The median number of enrolled ICU patients was 11,425 (interquartile range, 1,020 to 30,906). Nineteen studies targeted specialized ICU populations, with neonates representing the most frequently studied groups (*n* = 3; Fig. A2), whereas the remaining 67 studies did not specify a particular ICU setting.

### Data modalities and modeling approaches

**Fig. 5.**
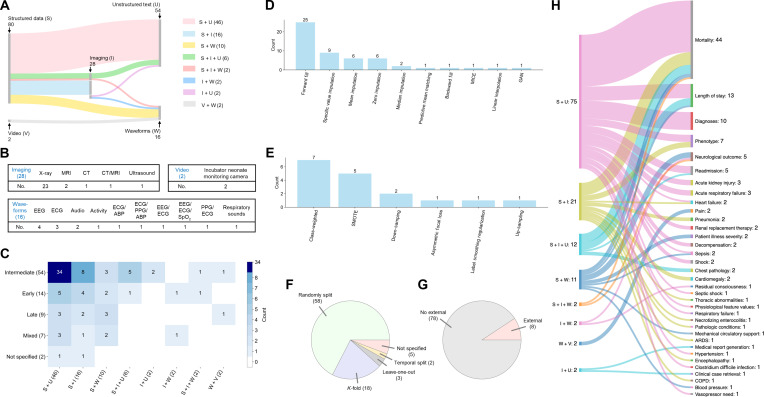
Summary of data modalities and modeling approaches used. (A) Utilized data modalities and their combinations. (B) Specific data utilized in imaging, waveforms, and video modalities. (C) Distribution of modality combinations and their corresponding fusion methods. (D) Missing data imputation methods for structured data modality (note count refers to the total frequency of occurrence across included studies, where multiple methods may be reported within a single study). (E) Methods for addressing class imbalance. (F) Dataset partitioning schemes. (G) Number of external validation. (H) Distribution of clinical task topics. ABP, arterial blood pressure; ARDS, acute respiratory distress syndrome; CT, computed tomography; COPD, chronic obstructive pulmonary disease; EEG, electroencephalogram; ECG, electrocardiogram; GAN, generative adversarial network; MRI, magnetic resonance imaging; MICE, multivariate imputation by chained equations; PPG, photoplethysmography.

As shown in Fig. 5A, a total of 5 modalities were identified across the 86 studies, with structured data (“S”; *n* = 80) and unstructured text (“U”; *n* = 54) being the most commonly used modalities, followed by imaging (“I”; *n* = 28), waveforms (“W”; *n* = 16), and video (“V”; *n* = 2). No included multimodal studies utilized omics data. Among studies include imaging modality, x-ray was predominant (82%), whereas ECG was the most frequent waveforms source (50%); all video data were derived from incubator-mounted neonatal monitoring cameras (Fig. 5B). The most prevalent modality combinations were “S + U” (*n* = 46), “S + I” (*n* = 16), “S + W” (*n* = 10), and “S + I + U” (*n* = 6).

Intermediate fusion was the most commonly used modality fusion strategy (*n* = 54), particularly in “S + U” and “S + I” fusion, indicating a preference for deep feature-level representation fusion (Fig. 5C). In contrast, early (*n* = 14) and late (*n* = 9) fusion approaches were less commonly used. A smaller subset of studies (*n* = 7) adopted mixed fusion strategies, while 2 studies did not specify the fusion method applied.

An overview of 63 methods used for feature extraction before modality fusion and 21 methods applied for clinical outcome tasks following modality fusion is presented in Table 1 and Table A3. For structured data, unstructured text, and imaging modalities, the most commonly used techniques to derive feature representations from raw inputs were recurrent-neural-network-based methods, bidirectional encoder representations from transformers (BERT)-related models, and ResNet-related architectures, respectively. For the modality of waveforms, manually engineered features were most prevalent. In contrast, autoencoder-based approaches were predominantly utilized for processing video data. For the methods applied for specific downstream clinical tasks after fusion, XGBoost was the most frequently used model among studies using early fusion, while intermediate and mixed fusion studies mainly used fully connected layers. In contrast, late fusion approaches most often utilized weighted-sum aggregation to generate final output.

**Table 1.** Methods applied before modality fusion and following

Fusion strategy	Methods applied before modality fusion		Methods applied in downstream clinical tasks following modality fusion (count)^c^
	Modality^a^	Methods (count)^b^	
Early	S	Raw data (7), handcrafted features (5)	XGBoost (4), LR (3), SVM (2), AutoGluon (1), GBM (1), GRU (1), LR or SVM or RF or GBM (1), SVM or RF (1)
	U	Latent Dirichlet allocation (3), handcrafted features (2), frozen ClinicalBERT (1)	
	I	Handcrafted features (5), frozen MobileNet (1), frozen Densenet121-Res224-CheX (1)	
	W	Handcrafted features (4)	
Intermediate	S	LSTM (15), linear layer (10), CNN (8), MLP (5), GRU (3), Bi-LSTM (3), Transformer (3), Time2Vec (2), GCN (2), Encoder (2), T-transformer (1), piecewise linear encoding (1), RNN (1), perceiver (1), ConvTransformer (1), Bi-GRU (1), Self-supervised Transformer for time series (1), ordered neuron-LSTM (1)	Fully connected layer (35), MLP (12), LSTM (1), LSTM/GRU + fully connected layer (1), MLP + Cox (1), small language models (1), Bayesian inference (1), Qwen-1.5 32B (1), kNN (1)
	U	ClinicalBERT (7), CNN (7), BioBERT (5), Transformer (4), BlueBERT (2), TextEncoder (2), BioClinicalBERT (2), LSTM (2), Doc2Vec (2), Bi-LSTM (2), Bi-GRU (2), Word2vec (2), GCN (1), MLP (1), GloVec-CNN (1), IMUI-CNN (1), BioSent2Vec (1), Apache clinical text analysis (1), knowledge extraction system (1), Qwen-1.5 32B (1), Google-T5 (1), BioMedBERT (1), Longformer (1), Word2Vec (1), clinical BioBERT (1), GloVec (1), SapBERT (1), Cui2Vec (1), BioClincalBERT (1), named entity recognition (1), entity recognition (1)	
	I	CNN (3), ResNet (2), ResNet-50 (2), vision Transformer (2), ResNet-34 (2), ChexNet (1), ResNet34 (1), swin Transformer (1), Densenet121-Res224-CheX (1), LSTM (1), InceptionResnet-V2 (1)	
	W	CNN (2), MLP (1), Google’s VGGish (1), LSTM-based autoencoder (1), GRU (1), LSTM (1)	
	V	FaceNet-based autoencoder (1), Resnet18-based autoencoder (1), LSTM-based autoencoder (1)	
Late	S	XGBoost (3), LSTM (2), CNN (1), HIVE-COTE (1), GBM (1), TabTransformer (1), MLP (1), RF (1), LR (1), Decision tree (1)	Weighted sum (3), LR (2), Ensemble individual scores (1), SVM (1), unweighted average (1), unweighted majority voting (1)
	U	BioClincalBERT (1), Text-CNN (1), Char-CNN (1), Bi-LSTM (1)	
	I	DenseNet121 (1), Resnet-50 (1), vision Transformer (1)	
	W	Variational autoencoder (1), HIVE-COTE (1), CNN (1), RNN (1), VGG-16 (1), MLP (1), ResNeXt (1)	
	V	Bilinear CNN (1), VGG-16 (1), LSTM (1), MLP (1)	
Mixed	S	Raw data (3), handcrafted features (2), Transformer (1)	Fully connected layer (3), RF (2), LSTM + Transformer (1), Lasso regression (1)
	U	Transformer (2), ClinicalBERT (1), Bag-of-words (1)	
	I	CNN (1)	
	W	Handcrafted features (1), DenseNet (1), ridge regression classifier (1), GRU (1)	

^a^ S, structured data; I, imaging; W, waveforms; U, unstructured text; V, video.

^b^ Refers to the total frequency of occurrence across all included studies, where multiple methods may be reported within a single study.

^c^ Refers to the number of publications.

Bi, bidirectional; BERT, bidirectional encoder representations from transformers; CNN, convolutional neural network; EEG, electroencephalogram; ECG, electrocardiogram; GBM, gradient boosting machine; GCN, graph convolutional network; GRU, gated recurrent unit; HIVE-COTE, hierarchical vote collective of Transformation-based Ensembles; kNN, *k*-nearest neighbors; LSTM, long short-term memory; LR, logistic regression; MLP, multilayer perceptron; MRI, magnetic resonance imaging; SVM, support vector machine; PPG, photoplethysmogram; RF, random forest; RNN, recurrent neural network; VGG, visual geometry group; Vec, vector.

When constructing multimodal AI models, only 6 studies explicitly addressed the issue of missing modalities, referring to situations in which an entire modality was absent from the dataset (Table A4). Regarding missing values present within a given modality, 36 studies specifically proposed methods for imputing missing values in structured data modality, among which forward fill was the most commonly utilized approach (Fig. 5D and Table A5). Only one study explicitly addressed missing values in waveforms modality, using a combination of forward fill, specific value imputation, and mean imputation techniques. Similarly, just one study used resampling to reconstruct video data when faced with insufficient frames. No study discussed strategies for handling internal missing values within imaging or unstructured text data modalities. When addressing class imbalance, the most commonly used approach was class-weighted techniques (*n* = 7), followed by synthetic minority oversampling technique (SMOTE; *n* = 5), and downsampling methods (*n* = 2), as shown in Fig. 5E.

### Clinical tasks and model performance

Most studies evaluated model performance using random splitting (*n* = 58) and *K*-fold cross-validation (*n* = 18), followed by leave-one-out (*n* = 3) and temporal split methods (*n* = 2). Five studies did not specify the approach used for dataset partitioning (Fig. 5F). In addition, only 8 studies performed external validation, underscoring a key limitation in the assessment of model generalizability (Fig. 5G). The correspondence between different modality combinations and topics of the 35 clinical tasks examined across the 86 included studies is shown in Fig. 5H. Mortality prediction was the most frequently investigated clinical task (*n* = 44), followed by prediction of length of stay (*n* = 13), diagnostic prediction (*n* = 10), and phenotype classification (*n* = 7). The majority reported outcome prediction tasks without explicit specification of corresponding clinical interventions. Only 4 studies specified predictions related to clinical interventions, including early prediction of vasopressor requirement, initiation of continuous renal replacement therapy, mechanical circulatory support, and dialysis need.

**Fig. 6.**
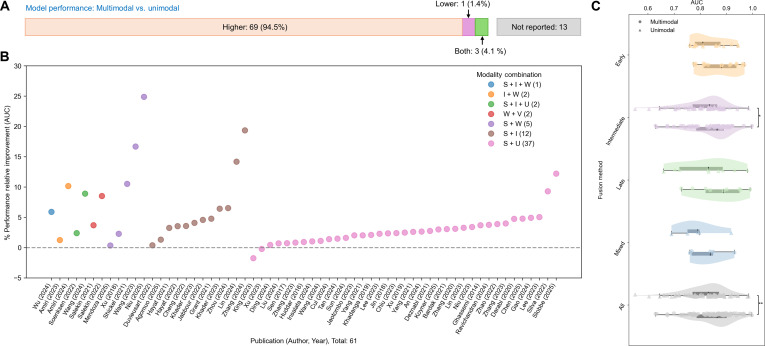
Performance comparison between multimodal and unimodal models. (A) Number of publications that explicitly report comparative performance outcomes (superior or inferior) for multimodal versus unimodal models. (B) Distribution of relative area under the curve (AUC) performance improvement across the 61 studies reporting AUC metrics. (C) AUC performances of multimodal and unimodal approaches under different fusion methods. Statistical significance was assessed using a 2-sample *t* test with unequal variances; *P < 0.05 and **P < 0.01. I, imaging; S, structured data; U, unstructured text; V, video; W, waveforms.

A total of 73 studies compared the performance of multimodal versus unimodal approaches. Of these, 69 (94.6%) reported superior performance with multimodal methods, whereas one study (1.4%) found inferior performance, and another 3 (4.1%) reported mixed results (Fig. 6A). The distribution of relative performance improvements among the 61 studies reporting AUC metrics is shown in Fig. 6B, with a mean AUC relative improvement of 4.4% (95% CI: 3.2 to 5.7). The AUC performances of multimodal and unimodal approaches under different fusion methods are shown in Fig. 6C. Table A6 summarizes 5 studies comparing multimodal models and clinicians, showing that multimodal models perform comparably to or better than clinicians, with overall AUCs of 0.83 to 0.84 for the models versus 0.79 to 0.82 for clinicians.

Of the 86 studies reviewed, 32 applied explainability techniques to explain model outputs, and 14 distinct explainability methods were identified. The most frequently utilized methods included attention mechanisms (*n* = 10), SHapley Additive exPlanations (SHAP; *n* = 9), integrated gradients (*n* = 4), and Gradient-weighted Class Activation Mapping (Grad-CAM; *n* = 4) (Fig. A3).

### Transparency and reproducibility

Transparency and reproducibility are fundamental principles underlying rigorous scientific research. However, among the 86 studies reviewed, explicit adherence to established guidelines was infrequently reported: Only 2 studies acknowledged compliance with the Transparent Reporting of a multivariable prediction model for Individual Prognosis or Diagnosis (TRIPOD) guidelines, and one study followed the TRIPOD+AI guideline (Table A7). Regarding reproducibility, 34 studies provided publicly accessible implementation codes (Table A1). In addition, only 9 open-source multimodal ICU databases were identified from the included studies (Table 2).

**Table 2.** Identified open-source multimodal ICU database from included studies

First released year	Database	Data source	Patient type	No. of patients	Modality type^a^	Link source
2018	eICU-Collaborative Research Database (eICU-CRD)	335 ICUs at 208 hospitals located throughout the US (2014–2015)	Overall	139,367	S: Time-invariant and time-variant variables	https://physionet.org/content/eicu-crd/2.0/
					U: Diagnosis, medication, etc. in text type	
2021	University of South Florida Multimodal Neonatal Pain Assessment Dataset (USF-MNPAD-I)	Neonatal ICU, Tampa General Hospital, FL, USA	Neonates	58	S: Vital signs, contextual and medical data	https://rpal.cse.usf.edu/project_neonatal_pain/dataset.html
					W: Audio	
					V: Incubator neonate monitoring camera	
2023	International Cardiac Arrest Research consortium database (I-CARE)	ICU in 1 of 7 academic hospitals in the US and Europe	Comatose patients following cardiac arrest	607	S: Baseline clinical variables	https://physionet.org/content/i-care/2.1/
					W: EEG, ECG	
2023	CoCross COVID-19 Lung Sounds Database	The 1st ICU of “G. Papanikolaou” hospital in Thessaloniki, Greece	COVID-19	171	S: Clinical variables	https://figshare.com/s/e5af036d5ca46150eac4
					I: X-ray	
					W: Respiratory sounds	
	MIMIC series					
2011	MIMIC-II	Beth Israel Deaconess Medical Center in Boston, MA, USA (2001–2007)	Overall	22,870	S: Time-invariant and time-variant variables	No longer available
					U: Free-text clinical notes	
2015	MIMIC-III	Beth Israel Deaconess Medical Center in Boston, MA, USA (2001–2012)	Overall	46,520	S Time-invariant and time-variant variables	https://physionet.org/content/mimiciii/1.4/
					U: Free-text clinical notes	
2019	MIMIC Chest X-ray (MIMIC-CXR)	Beth Israel Deaconess Medical Center in Boston, MA, USA (2011–2016)	Overall	65,379	I: X-ray	https://physionet.org/content/mimic-cxr/2.1.0/
					U: Free-text radiology reports	
2020	MIMIC-III Waveform Matched Subset	Beth Israel Deaconess Medical Center in Boston, MA, USA (link to MIMIC-III)	Overall	10,282	W: ECG, ABP, respiration, PPG, etc.	https://physionet.org/content/mimic3wdb-matched/1.0/
2020	MIMIC-IV	Beth Israel Deaconess Medical Center in Boston, MA, USA (2008-2022)	Overall	50,920	S: Time-invariant and time-variant variables	https://physionet.org/content/mimiciv/3.1/
					U: Free-text clinical notes	

^a^ S, structured data; I, imaging; W, waveforms; U, unstructured text; V, video.

ABP, arterial blood pressure; EEG, electroencephalogram; ECG, electrocardiogram; ICU, intensive care unit; MIMIC, Medical Information Mart for Intensive Care; PPG, photoplethysmogram.

## Discussion

To the best of our knowledge, this scoping review is the first to systematically map the landscape of multimodal AI applications in critical care. A total of 86 studies published between 2010 January and 2025 April were included in the final analysis. A substantial growth in this field was observed, with 85% published within the past 5 years, reflecting heightened research interest and advancements in integrating multimodal data for precision medicine in the ICU. Across the board, multimodal AI models demonstrated superior performance compared to unimodal approaches, achieving a mean relative AUC improvement of 4.4% (95% CI: 3.2 to 5.7), thereby highlighting the promise of multimodal approaches to enhancing diagnosis or prognosis prediction accuracy in critical care settings.

### Benefits and landscape of multimodal AI

The primary advantage of multimodal AI lies in its ability to integrate diverse data modalities, thus leveraging complementary information across modalities. In this review, most studies found that multimodal models outperformed unimodal models, which is consistent with real-world clinical practice, where information from different sources is usually combined rather than considered separately. Only Fang et al. [34] did not report superior performance with multimodal models, although they did highlight increased robustness. Overall, our findings align with previous reviews: Huang et al. [23] reported an increase in predictive performance (2% to 16% AUC improvement) for multimodal fusion of medical imaging and EHR data; similarly, Kline et al. [17] observed a mean AUC improvement of 6.4% when applying multimodal machine learning in precision health; and Mohsen et al. [35] identified an average AUC increase of 7% using multimodal fusion methods for diabetes risk prediction. In critical care settings, the observed 4.4% relative improvement in AUC reflects better discrimination than baseline models and represents a clinically meaningful improvement for risk stratification in high-risk ICU cohorts. However, performance gains alone may not readily translate into clinical benefit. Their significance is more likely when model outputs are linked to concrete clinical decisions or interventions, rather than evaluated solely using discrimination metrics. In the current literature, most multimodal AI studies in critical care focus on outcome prediction, particularly mortality and length of stay. While useful for benchmarking, these tasks have limited clinical impact when not explicitly linked to clinical actions or care pathways. Only a small number of studies connected multimodal predictions to clinical interventions [36–39], underscoring a gap between methodological innovation and bedside decision-making. Addressing this gap will be critical for advancing multimodal AI from proof-of-concept models to clinically meaningful decision-support tools in precision critical care.

When using multimodal AI, it is essential to identify optimal fusion strategies for integrating multimodal data that effectively capture relationships between each modality and clinical outcomes. In this review, intermediate fusion emerged as the most frequently used strategy, as it effectively integrates multimodal information within the latent feature space of deep learning architectures by automatically uncovering and modeling complementary intermodal relationships through loss-function backpropagation, thereby potentially enhancing the richness of feature representations and improving model accuracy [7]. While simple concatenation of deeply extracted features remains the most straightforward approach, attention mechanisms such as gated attention [40,41], cross-attention [42–45], or transformer encoders [20,36,46–49] are increasingly adopted. These mechanisms dynamically capture and weight global cross-modal interactions, yielding richer feature fusion compared to straightforward concatenation [7]. However, intermediate fusion approaches typically involve higher complexity and computational costs, and the black-box nature of deep learning architectures limits their clinical explainability. Early fusion was the second most commonly used strategy in this review. It primarily combines raw data or handcrafted features with traditional machine learning models for clinical tasks, enabling joint representation learning. However, it requires precise modality alignment and struggles when modalities are temporally or semantically disjoint. Compared with intermediate fusion, this strategy offers greater clinical acceptability because its modality representations are grounded in clinical prior knowledge and are thus more readily explainable by clinicians. Late fusion aggregates the outputs of independently trained unimodal models, providing robustness to missing data and making it easier to integrate new modalities compared to early or intermediate fusion. However, it is limited in its ability to capture interactions between modalities. Mixed fusion offers flexible integration of different modalities, which helps address modality imbalances. However, its design is challenging because it requires careful consideration of when and how to combine modalities [7], making it the least commonly used fusion method in this review. Given that the reviewed strategies vary widely in target populations, sample sizes, methodologies used before and after fusion, and measured clinical outcomes, identifying a universally optimal fusion strategy remains challenging. Table 3 summarizes the characteristics of different modality fusion strategies utilized in multimodal AI for critical care, providing a roadmap for further researchers to select the approach best suited to their clinical context.

**Table 3.** Comparison of different strategies for modality fusion

Attribute	Early fusion	Intermediate fusion	Late fusion
Training data demand	Low	High	Medium (depending on the unimodality model)
Model design complexity	Low	High	Medium (depending on the unimodality model)
Multiple models training	No	Yes	Yes
Training computational overhead	Low	High	Medium (depending on unimodality model)
Cross-modal interaction modeling	Yes	Yes	No
Cross-modal data alignment	Possible with handcrafted features or frozen pretrained model	Yes	No
Missing modality handling	No	Possible with specialized architectures	Yes
Fusion timing flexibility	Low (fixed fusion at raw input)	High (fuse at different feature representation levels)	Low (fixed fusion at decision level)
Modality scalability	Low	Medium	High
Data noise robustness	Low	Medium	High
Clinical interpretability	High (clinical knowledge usually guides feature engineering)	Low (black-box property of deep neural networks)	Medium (easiest to determine key modality; but for model explainability, depending on the unimodality model)

In multimodal ICU modeling, different data types are collected at different temporal scales, such as continuously monitored physiological waveforms and less frequently updated or static EHR variables, leading to temporal misalignment across data sources. Across the reviewed studies, how this misalignment was handled largely depended on the fusion strategy. Early fusion approaches typically aggregated features within predefined time windows for joint modeling [50–53], whereas late fusion combined outputs from modality-specific models without requiring explicit data-level alignment [39,54–56]. Intermediate fusion methods modeled each modality separately over time before integrating learned representations using concatenation [57,58] or attention [18,59], but most relied on retrospective alignment within relatively coarse time windows. In practice, multimodal data were commonly aligned to clinically meaningful reference points, such as first day after ICU admission or predefined prediction horizons, with static or sparsely sampled variables appended to time-resolved data. Although these pragmatic strategies facilitate multimodal integration in real-world ICU settings, they involve trade-offs between temporal resolution and information loss, underscoring the need for temporally aware alignment approaches that go beyond coarse windowing by structurally decoupling time-aligned and asynchronous modalities at the model level [60].

Collecting and curating large-scale annotated datasets that encompass a diverse range of modalities is crucial for the development of multimodal data-driven AI models in critical care. In this review, we found that structured data and unstructured text are the most frequently utilized modalities, likely due to their widespread availability in the ICU, ease of storage and processing, and broad applicability across a wide range of clinical tasks. Although imaging and waveforms data are the next most commonly used modalities, they remain underutilized in ICU multimodal AI due to high storage requirements and their primary relevance to specific clinical tasks, such as neurological outcome prediction with EEG [19,52,53,56,61] or MRI [19,52], pneumonia detection via chest x-ray [62,63], and forecasting mechanical circulatory support needs from ECG and ABP [39]. In addition to the types of waveform and imaging data summarized in Fig. 5B, intracranial pressure monitoring in neurointensive care [64,65], near-infrared spectroscopy for regional tissue oxygenation assessment [66,67], and infrared thermography imaging for peripheral perfusion evaluation in patients with septic shock [68] have been applied effectively. Investigating how these data can be combined with other modalities in multimodal AI models to improve clinical performance offers a promising direction for future research. Regarding video data, only 2 studies in this review used camera recordings to assess postoperative pain in neonates by capturing movements in premature infants [55,69]. Although video data use in the ICU has been limited by storage demands and patient privacy concerns, its application in vital signs estimation [70–72] and event or activity monitoring [73] is growing, underscoring the broader potential for integrating video modality into multimodal AI frameworks for critical care applications. In addition, although omics data have been used in unimodal AI models for the ICU [74–76], they were absent from the multimodal studies included in this review. In oncology [27] and cancer biomarker discovery [26], integrating omics within multimodal AI frameworks has demonstrated improved performance, suggesting that omics data hold promise for exploration in future multimodal AI applications in critical care. However, omics data are not yet routinely or rapidly available at the bedside, which may hinder their immediate integration into real-time clinical decision-making. Nevertheless, despite recognizing the potential of diverse multimodal data, practical constraints on labeled dataset size and availability pose substantial challenges.

To address the common challenge of limited labeled dataset availability at the initial stage of applying multimodal AI to new critical care tasks targeting specific patient cohorts, this review outlines several potential strategies to mitigate this issue. One effective strategy is to use pretrained models that have learned generalizable features from large and diverse datasets, followed by fine-tuning them on the target clinical data. This approach enables efficient adaptation to specialized clinical tasks, even with limited data from the specific patient cohort [77]. Specifically for unstructured text data, several pretrained models, such as BlueBERT [21,78] and BioClinicalBERT [42,43,79,80] (trained on PubMed and MIMIC-III database [81]), BioBERT [82–85] and BioMedBERT [37] (trained on PubMed database), ClinicalBERT [40,41,45,47,49,86–88] (trained on MIMIC-III database [81]), and SapBERT [87] (trained on the Unified Medical Language System database [89]), have been used to enhance representation learning for downstream clinical tasks. In addition, large language models (LLMs), such as Qwen-1.5 [46] and Google-T5 [90], have also been leveraged in clinical multimodal studies. Emerging evidence suggests that multimodal LLMs may exhibit more consistent performance across demographic subgroups, indicating potential advantages in fairness compared with some traditional models [91]. For imaging modalities, domain-specific architectures such as CheXNet [21] and MobileNet [62] pretrained on the ChestX-ray14 dataset [92], DenseNet-121 [93], ResNet-34 [94] and ResNet-50 [95] pretrained on ImageNet [96], and DenseNet121-Res224-CheX [97] pretrained on the CheXpert dataset [98] have been used to enhance deep feature representation for imaging data. For waveforms data, Google’s VGGish [69] model pretrained on the YouTube-8M dataset [99] has been applied to extract audio embeddings for neonatal postoperative pain estimation. Another helpful strategy is self-supervised pretraining, a technique in which models learn to extract meaningful patterns from unlabeled data by generating knowledge based on the data itself, allowing more effective utilization of available raw data across various modalities [100]. For example, King et al. [101] first used a self-supervised pretraining framework combining contrastive and masked token prediction to align clinical notes with structured data and then reporting a 17% increase in AUC for in-hospital mortality prediction when only 1% of labels were available for training. Ding et al. [90] and Fang et al. [34] also used contrastive learning to capture cross-modal correlations within individual patients for health event prediction and clinical case retrieval, respectively, without relying on labeled data. In addition, although not identified by the studies included in this review, medical multimodal foundation models [102] are emerging as a promising strategy and align with the paradigm of Generalist Medical AI [103]. This paradigm reflects a shift from task-specific medical AI toward large-scale foundation models that learn unified representations across heterogeneous modalities, including text, waveforms, images, and structured EHRs, through large-scale pretraining. Within this framework, multimodal foundation models can be fine-tuned or directly applied to a wide range of downstream clinical tasks in critical care. For example, Hamamci et al. [104] developed a vision-language 3-dimensional medical imaging foundational chat model to support multiabnormality detection and case retrieval; Tian et al. [105] developed a knowledge-enhanced automated ECG diagnostic system capable of providing explanations for any form and rhythm identified on ECG; and Zhang et al. [106] proposed a vision-language foundation model applicable to diverse biomedical tasks, such as mortality prediction, treatment suggestion, and radiology report generation. Compared with traditional pretrained unimodal models, multimodal foundation models aligned with the Generalist Medical AI paradigm provide greater adaptability by integrating multiple sources of clinical information. Therefore, developing large-scale multimodal foundation models for ICU settings represents an important step toward realizing Generalist Medical AI in critical care.

### Barriers and recommendations

**Fig. 7.**
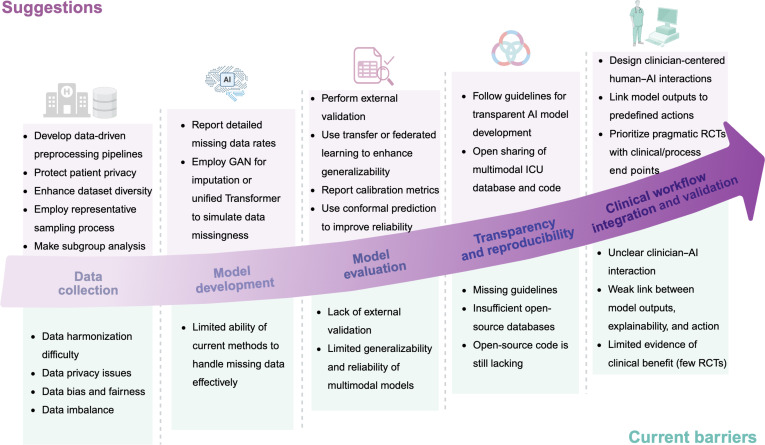
Summary of current barriers and suggestions. AI, artificial intelligence; GAN, generative adversarial network; ICU, intensive care unit; RCT, randomized controlled trial.

Although multimodal AI models for critical care have advanced considerably, this scoping review identifies some barriers that limit both research progress and clinical application (Fig. 7). The first barrier concerns challenges in data collection, including difficulties in data harmonization, issues of data privacy, as well as data bias and imbalance. Multimodal data harmonization is particularly challenging, as differences in data formats, medical devices, variable definitions, and measurement units across centers make it difficult to effectively combine and analyze diverse datasets for robust model development and validation [8,107]. Data privacy and sharing concerns further complicate the process, as integrating information from multiple sources must comply with strict regulations and protect sensitive patient data. We recommend developing modality-specific or integrated data-driven preprocessing pipelines, such as the open-source Flexible Data-Driven Pipeline framework [108], which streamlines preprocessing of EHR data, to address these challenges in multimodal research. In multimodal AI, variations in clinical practice, resource availability, and patient characteristics can lead to systematic underrepresentation of certain patient groups or clinical scenarios in the data, resulting in biased model training and reduced fairness [8,17]. In addition, data imbalance occurs when certain groups or classes are over- or underrepresented in a dataset, which can introduce bias into the performance of AI models [17]. Therefore, we recommend following the standards proposed by Arora et al. [109] for health dataset use in AI applications: implement clear, standardized data collection protocols that harmonize definitions and categories, use representative sampling across key demographic and clinical subgroups to ensure dataset diversity, and provide comprehensive documentation of preprocessing steps, consent procedures, and inclusion and exclusion criteria to ensure comparability and minimize bias and imbalance. In addition, in practical clinical applications, missing data remain a common challenge, including both missing modalities and missing values within a single modality.

The second barrier concerns the insufficient capability of current methods to effectively handle missing data during model development. In this review, only 6 studies addressed the issue of missing modalities [20,48,69,110–112]. Regarding missing values within individual modalities, 36 studies focused on structured data, with only one study each considering waveforms [39] and video modality [55]. We recommend that the issue of missing data be given careful consideration during the development of multimodal models. Appropriate strategies should be adopted to address this challenge, such as using deep generative models [69,84] for data imputation or leveraging approaches that simulate data missingness within unified Transformer architectures [20,48].

The third barrier is the limited generalizability and reliability of multimodal models. In this review, only 8 studies performed independent external validation [38,41,42,46,63,93,113,114], revealing an average 3.5% decrease in AUC on external datasets. This is likely attributable to the heterogeneity of medical data across institutions, resulting in distributional shifts that undermine model generalizability [115]. Integrating multiple data modalities may further exacerbate this issue by increasing complexity and sources of variability. We recommend that external validation be performed and reported to assess model generalizability. Furthermore, techniques such as transfer learning [116,117] and federated learning [118,119] are encouraged to improve the robustness and generalizability of multimodal models, with federated learning also protecting data privacy. In addition, only 5 studies reported calibration metrics, which are crucial for assessing model reliability [38,63,113,120,121]. Therefore, we recommend increased reporting of calibration metrics and consider using conformal prediction methods to enhance model reliability [122,123].

The fourth barrier is insufficient transparency and reproducibility in the existing literature. This review reveals that open-source multimodal ICU databases identified from the included studies remain scarce. Although we manually searched 7 additional open-source multimodal ICU databases from websites or PhysioNet [124], most of these predominantly contain structured data and unstructured text (Table A8). The diversity of data modalities available in these databases remains limited. Furthermore, only approximately 40% of the reviewed studies reported publicly accessible implementation code. We recommend following appropriate guidelines to improve transparency when developing different AI models. For example, the TRIPOD+AI statement [125] provides reporting guidance for machine-learning-based diagnosis or prognosis prediction models, the TRIPOD-LLM [126] reporting guideline for using LLMs, and the CONSORT-AI [127] for AI-based clinical trials. In addition, we encourage the open sharing of more diverse multimodal ICU databases and model implementation code to enhance reproducibility.

The fifth barrier is the insufficient translation of multimodal AI models into clinical practice, which represents a central challenge for precision critical care. Although 14 different explainability methods have been applied to these models, interpretability in critical care should be considered at 2 levels: technical explainability (how the model arrives at a prediction) versus clinical interpretability (whether the explanation is understandable, trustworthy, and supports action by intensivists). Common outputs such as Grad-CAM heatmaps or SHAP plots may not be intuitively interpretable in time-pressured ICU settings and, by themselves, may not justify why clinicians should trust or act on a model prediction. Moreover, clinician–AI interaction is rarely evaluated: Existing comparisons are often framed as model-versus-clinician rather than testing clinician-with-model versus clinician-alone performance, and no studies assess whether explanations improve decision quality or efficiency without increasing cognitive burden or automation bias. Beyond explainability, effective clinical integration also depends on how model outputs are embedded into real-world workflows; most studies do not specify whether predictions are delivered as interruptive alerts or integrated dashboards nor how alert fatigue is mitigated in information-dense environments. Model outputs are frequently presented as risk scores without clearly defined links to actionable clinical interventions, limiting bedside utility. Successful integration therefore relies not only on technical performance but also on interoperability with existing clinical information systems, well-defined decision pathways that connect predictions to actions, and human-centered evaluation designs [128,129]. Importantly, while prospective validation is frequently recommended, observational validation alone is insufficient to establish clinical benefit. Randomized clinical trials remain essential to determine whether multimodal AI-assisted workflows improve patient-centered outcomes or key care processes compared with standard-of-care practice.

### Limitations

There are several limitations to this scoping review. First, although our search strategy was comprehensive, some relevant studies may still have been missed. We did not include non-English publications, which could have caused language bias and led to missing important research from other regions. In addition, some relevant studies may have been overlooked because of variations in terminology and the rapidly evolving nature of this research area. Second, the included studies showed considerable heterogeneity regarding ICU patient populations, data modalities, AI models, and clinical outcomes. This variability complicated direct comparison and hindered the synthesis of findings, thereby limiting our ability to draw broadly generalizable conclusions. Third, we did not perform a formal critical appraisal or risk of bias assessment of the included studies, consistent with the methodology of scoping reviews. As a result, the overall reliability and validity of the synthesized evidence may be limited, particularly considering the likely heterogeneity in methodological rigor across studies. Nevertheless, we believe that the evidence summarized in this review provides a reference framework for guiding future research and facilitating the development of reliable, clinically useful multimodal AI tools in critical care.

## Conclusion

In conclusion, our scoping review provides a comprehensive overview of multimodal AI approaches in critical care, highlighting their growing interest and promising potential. Multimodal models demonstrated superior predictive performance compared to unimodal methods by effectively integrating heterogeneous clinical data. However, challenges remain, including data harmonization, privacy issues, bias and imbalance, missing data, model generalizability, reliability, transparency, and clinical translation. Rigorous validation through clinical trials and prospective studies is essential to confirm the reliability and clinical utility of these models. Ultimately, the effective integration of multimodal AI into clinical practice will require close collaboration between clinicians and AI developers so that these technologies can contribute to improving patient outcomes and supporting clinical decision-making.

## Data Availability

All data generated or analyzed during this study are included in the Supplementary Materials.

## Supplementary Materials

Supplementary 1 Tables A1 to A8 Figs. A1 to A3
