# Supplementary material for: Multimodal Artificial Intelligence for Precision Critical Care: A Scoping Review
Source: Health Data Sci. 2026 Apr 16;6:0356. doi: 10.34133/hds.0356 (PMC13084062; doi:10.34133/hds.0356)
Supplement: Supplementary 1 — Tables A1 to A8 Figs. A1 to A3 [file hds.0356.f1.docx]

**Supplementary Materials**

Appendix Table 1. ﻿Search strategy for studies reported multimodal machine learning in critical care

| Database | Terms | Results |
| --- | --- | --- |
| PubMed | (("critical care"[All Fields] OR "intensive care"[All Fields] OR "ICU"[All Fields] OR "critical illness"[All Fields] OR "critically ill"[All Fields] OR "acute care"[All Fields]) AND ("machine learning"[All Fields] OR "deep learning"[All Fields] OR "artificial intelligence"[All Fields] OR "AI"[All Fields] OR "neural network*"[All Fields]) AND ("multimodal"[All Fields] OR "multi-modal"[All Fields] OR "multiple modalities"[All Fields] OR "multiple data"[All Fields] OR "multi-view"[All Fields] OR "multi-source"[All Fields] OR "cross-modal"[All Fields] OR "data fusion"[All Fields]))  Filters: (Results by year >= 2010 AND <= 2025) AND (Article Language = "English") | 276 |
| Embase | ("critical care" OR "intensive care" OR ICU OR "critical illness" OR "critically ill" OR "acute care") AND ("machine learning" OR "deep learning" OR "artificial intelligence" OR AI OR "neural network*") AND ("multimodal" OR "multi-modal" OR "multiple modalities" OR "multiple data" OR "multi-view" OR "multi-source" OR "cross-modal" OR "data fusion")  Limits: (Publication Year: 2010 - Current) AND (English Language) | 189 |
| Scopus | TITLE-ABS-KEY (("critical care" OR "intensive care" OR "ICU" OR "critical illness" OR "critically ill" OR "acute care") AND ("machine learning" OR "deep learning" OR "artificial intelligence" OR "AI" OR "neural network*") AND ("multimodal" OR "multi-modal" OR "multiple modalities" OR "multiple data" OR "multi-view" OR "multi-source" OR "cross-modal" OR "data fusion")) AND PUBYEAR > 2009 AND PUBYEAR < 2026 AND ( LIMIT-TO ( LANGUAGE , "English")) | 239 |
| Web of Science | ALL fields (("critical care" OR "intensive care" OR ICU OR "critical illness" OR "critically ill" OR "acute care") AND ("machine learning" OR "deep learning" OR "artificial intelligence" OR AI OR "neural network") AND ("multimodal" OR "multi-modal" OR "multiple modalities" OR "multiple data" OR "multi-view" OR "multi-source" OR "cross-modal" OR "data fusion"))  Filters: (Publication Years >= 2010 AND <= 2025) AND (Languages = "English") | 220 |
| IEEE Xplore | ("All Metadata":"critical care" OR "All Metadata":"intensive care" OR "All Metadata":ICU OR "All Metadata":"critical illness" OR "All Metadata":"critically ill" OR "All Metadata":"acute care") AND ("All Metadata":"machine learning" OR "All Metadata":"deep learning" OR "All Metadata":"artificial intelligence" OR "All Metadata":AI OR "All Metadata":"neural network*") AND ("All Metadata":"multimodal" OR "All Metadata":"multi-modal" OR "All Metadata":"multiple modalities" OR "All Metadata":"multiple data" OR "All Metadata":"multi-view" OR "All Metadata":"multi-source" OR "All Metadata":"cross-modal" OR "All Metadata":"data fusion")  Filters: Year Range from 2010 to 2025 | 99 |

Appendix Table 2. Study Characteristics of included 86 studies

| **Ref.** | **Author (Year)** | **Data Source** | **Number of Patients** | **Modality ^a^** | **Fusion Method**  **(open-source code)** | **Uni-modal Method before Fusion ^a^** | **Model after/for Fusion** | **Clinical Outcome** | **Validation Processes** | **Explainable Method** | **Multimodal vs Uni-modal Performance (primary metric AUC) ^b^** |
| --- | --- | --- | --- | --- | --- | --- | --- | --- | --- | --- | --- |
| ^1^ | ﻿Niu (2025) | ﻿International Cardiac Arrest Research Consortium (I-CARE) database | 277 | S  W: EEG, ECG | Early  (No) | S: Raw features W: Handcrafted features | LR or SVM or RF or GBM | ﻿Neurological outcomes (good and poor) | K-fold cross validation | SHAP | 24-hour time window: 0.969 vs 0.776 |
| ^2^ | Kim (2025) | Seoul National University Hospital | 390 | S  I: CT, ﻿MRI  W: EEG | Early  (Yes) | S: Raw features W: Handcrafted features I: Handcrafted features | ﻿XGB | ﻿Neurological outcomes after cardiac arrest (good and poor) | Randomly split | SHAP | 0.965 (no comparison) |
| ^3^ | ﻿Mendoza (2025) | Texas Children’s Hospital | 106 | S:  W: ECG, PPG, ABP | Late  (No) | S: Raw features + XGB; Handcrafted features + LSTM/CNN/﻿HIVE-COTE W: VAE + HIVE-COTE | Unweighted average | Mechanical circulatory support need | K-fold cross validation | Gain criteria, Permutation feature importance | 0.889 vs 0.886 |
| ^4^ | Lin (2025) | MIMIC-IV; MIMIC-CXR | 9928 | S:  I: Chest X-ray U | Intermediate  (Yes) | S: ﻿Handcrafted features + MLP I: ﻿ChexNet + MLP U: ﻿BlueBERT + GCN/MLP | MLP | ICU mortality | Randomly split | NA | ﻿C-index: 0.783 vs 0.775 |
| ^5^ | ﻿Agomuo (2025) | MIMIC-IV;  ﻿MIMIC-CXR | NA | S  I: Chest X-ray | Early + Intermediate  (No) | S: ﻿Handcrafted features I: CNN | LSTM + Transformer | ﻿ICU mortality | NA | SHAP | 0.930 vs 0.918 |
| ^6^ | Chen (2025) | MIMIC-III | ﻿39,429 | S  U | Intermediate  (No) | S: CNN; T-transformer time-aware self-attention U: ClinicalBERT | ﻿Fully connected layer | ﻿In-hospital mortality | Randomly split | NA | 0.923 vs 0.881 |
| ^7^ | Slobbe (2025) | MIMIC-III | ﻿32,664 | S  U | Intermediate  (Yes) | S: CNN U: ﻿GloVe-CNN (GloVe embedding) + ﻿﻿IMUI-CNN (Clinical concepts from the Elsevier H-Graph) | ﻿Fully connected layer | ﻿AKI | K-fold cross validation | NA | 0.874 vs 0.779 |
| ^8^ | ﻿Fang (2025) | ﻿MIMIC-CXR | NA | I: Chest X-ray  U | Intermediate  (No) | I:﻿ CXR-Predictor (CNN) U: ﻿BioSent2Vec (﻿Sent2vec) | kNN | ﻿Clinical case retrieval | Randomly split | NA | ﻿The retrieval precision of the retrieved k cases (precision@k)  k=100: 77.9 vs 79.4 |
| ^9^ | Guo (2025) | MIMIC-IV;  ﻿MIMIC-CXR | ﻿10,804 | S  I: Chest X-ray | Intermediate  (No) | S: ﻿Two stacked LSTM I: ResNet34 | ﻿Fully connected layer | ﻿1: Phenotype classification (﻿25 types) 2: ﻿In-hospital mortality | Randomly split | NA | 1: 0.741 (no comparison) 2: 0.836 (no comparison) |
| ^10^ | ﻿Koyner (2025) | University of Wisconsin-Madison (2009-2020)  University of Chicago Medicine (2008-2022) | ﻿Chicago: 219,353 Wisconsin: 205,226 | S  U | Intermediate  (No) | S: ﻿Piecewise Linear Encoding + Liner layer; ﻿Time2Vec layer U: ﻿Apache clinical Text Analysis and Knowledge Extraction System | LSTM/GRU + ﻿Fully connected layer | ﻿1: Moderate to severe AKI 2: RRT | Temporal split | Integrated gradients | 1. 0.85 vs 0.83 2. 0.87 vs 0.84 |
| ^11^ | Liu (2025) | MIMIC-IV; MIMIC-CXR | ﻿377,110 (not patients) | I: Chest X-ray  U | Intermediate  (Yes) | ﻿I:﻿ Swin Transformer  U: Qwen-1.5 32B | Qwen-1.5 32B | ﻿Medical report generation | Randomly split | NA | ﻿MetaGP excels in generating medical imaging reports, producing high-quality outputs for chest X-rays |
| ^12^ | Liu (2024) | MIMIC-III | 31,965 | S U | Intermediate  (No) | S: CNN + ﻿Time2Vec U: ﻿TextEncoder | ﻿Fully connected layer | ﻿1: 48-hour in-hospital mortality prediction 2: 48-hour phenotype classification | Randomly split | NA | 1: 0.901 vs 0.893 2: 0.836 vs 0.832 |
| ^13^ | Ding (2024) | MIMIC-III | 7125 | S  U | Intermediate  (No) | S: ﻿CNN U: Google T548 as LLM | ﻿Fully connected layer | 1: ﻿Hypertension prediction 2: ﻿Heart failure prediction | Randomly split | Attention | 1: 0.781vs 0.778 2: 0.﻿853 vs 0.﻿849 |
| ^14^ | Wang (2024) | ﻿eICU, MIMIC-III (external validation but not mention No. of patients) | ﻿40,671 | S  U | Intermediate  (No) | S: LSTM; MLP; GCN U: BioClinical BERT | ﻿Fully connected layer | ﻿1: Mortality after 24h stay 2: ICU length of stay | Randomly split | Attention | 1: 0.922 vs 0.912 2: MAD 1.935 vs 2.276 External:  1: 0.964 2: 1.676 |
| ^15^ | Amiri (2024) | ﻿Rigshospitalet, Copenhagen University Hospital, Denmark | 45 | I: fMRI  W: EEG | Early + Late  (Yes) | W: Handcrafted features I: Handcrafted features | RF | ﻿3-month functional outcomes in ICU patients with acute disorders of consciousness | ﻿Leave-one-out cross validation | NA | 0.76 vs 0.69 |
| ^16^ | Sun (2024) | eICU | 24,600 | S  U | Intermediate  (No) | S: GCN U: ﻿BioClinicalBERT | MLP | ﻿1: ICU mortality 2: ICU ﻿  readmission | Randomly split | Attention | 1: 0.894 vs 0.888 2: 0.853 vs 0.832 |
| ^17^ | Gao (2024) | MIMIC-III; MIMIC-IV; eICU | MIMIC: 14,382 eICU: 7432 | S  U | Early + Intermediate  (No) | ﻿S: Handcrafted features U: Clinical BERT | ﻿Fully connected layer | ﻿Hospital mortality for ﻿heart failure patients | Randomly split | SHAP, Integrated gradients | Internal: 0.838 vs 0.790 Prospective: 0.849 vs 0.797 External: 0.767 vs 0.751 |
| ^18^ | Lin (2024) | Train: MIMIC-IV and MIMIC-CXR ﻿External: Department of Critical Care Medicine, First affiliated hospital of Soochow university | MIMIC: 3577 Soochow: 221 | S  I: Chest X-ray | Late  (Yes) | S: GBM I: ﻿DenseNet121 | LR | ﻿30-day mortality | Randomly split | SHAP, Partial dependence plots, Grad-CAM | Internal: 0.95 vs 0.83 External: 0.82 vs 0.72 |
| ^19^ | Tan (2024) | MIMIC-IV | ﻿45,127 | S  U | Intermediate  (No) | S: LSTM U: BioMedBERT | ﻿Fully connected layer | 1: AKI  2: CRRT | Randomly split | SHAP, Expected gradients | 1: 0.888 vs 0.873 2: 0.997 vs 0.985 |
| ^20^ | ﻿Mullie (2024) | MIMIC-IV; ﻿  MIMIC-CXR | 4000 | S  I: Chest X-ray | Intermediate  (Yes) | S: MLP I: CNN | ﻿Fully connected layer | ﻿In-hospital mortality | Randomly split | NA | ﻿0.820 (no comparison) |
| ^21^ | ﻿Tiwari (2024) | Local hospital | 143 | S  I: Chest X-ray | Early  (No) | S: NA I: ﻿MobileNet | ﻿GRU | Ventilator associated pneumonia early prediction | Randomly split | NA | 0.915 (no comparison) |
| ^22^ | ﻿Bjorkdahl (2024) | MIMIC-IV; ﻿MIMIC-CXR | ﻿14,854 | S  I: Chest X-ray  U | Intermediate  (Yes) | S: Encoder I: ﻿Densenet121-Res224-CheX U: ﻿Bio-BERT | ﻿Small Language Models | ﻿1: Chest pathology prediction 2: Prediction of the length of stay  3: 48h mortality prediction | Randomly split | NA | Recall and precision not always better than uni-modal |
| ^23^ | ﻿Saleh (2024) | MIMIC-III | 792 | S  U | Intermediate  (No) | S: RNN U:﻿ BioBERT-Transformer | ﻿Fully connected layer | ﻿ICU heart failure mortality prediction | Randomly split | NA | F1-score: ﻿91.693 vs 81.52 |
| ^24^ | ﻿Insalata (2024) | MIMIC-III | 14,216 | S  U | Late  (No) | ﻿S: TabTransformer U: BioClincalBERT + MLP | LR | ﻿In-hospital mortality | Randomly split | NA | 0.99 vs 0.98 |
| ^25^ | ﻿Ayden (2024) | MIMIC-III | ﻿33,330 | S  U | Intermediate  (No) | S: GRU + ﻿fully connected layer U: ﻿Longformer + fully connected layer | ﻿Fully connected layer | ﻿Automated ICD-9 code prediction | Randomly split | NA | Micro-F1: 0.69 vs 0.64 |
| ^26^ | ﻿Cui (2024) | Local hospital | ﻿408 | S I: ﻿Abdominal X-ray | Intermediate  (No) | S: CNN I: ResNet | ﻿Fully connected layer | Early diagnosis of necrotizing enterocolitis in newborns | Randomly split | Grad-CAM | F1-score: 0.9557 vs 0.8928 |
| ^27^ | ﻿Wu (2024) | ﻿G. Papanikolaou Hospital in Thessaloniki, Greece | 171 | S  I: Chest X-ray  W: ﻿Respiratory sounds | Intermediate  (No) | S: Fully connected layer W: MLP I: RenNet-50 + LSTM | ﻿Fully connected layer | ﻿1: ICU ﻿mortality for COVID-19  2: 90-day mortality | K-fold cross validation | NA | 1: 0.759 vs 0.718 2: 0.752 vs 0.709 |
| ^28^ | Zhang (2024) | Chifeng Municipal Hospital | 101 | S  I: Ultrasound | Early  (No) | S: Raw data I: Handcrafted features | LR | Sepsis-associated encephalopathy prediction | Randomly split | Coefficients | 0.938 vs 0.786 |
| ^29^ | Chen (2024) | MIMIC-IV; ﻿  MIMIC-CXR | NA | S  U  I: ﻿Chest X-ray | Intermediate  (No) | S: ﻿Attention-embedded 1D CNN U: LSTM I: ﻿InceptionResnet V2 | MLP | ﻿Inpatient length of stay | Randomly split | NA | ﻿MAE: ﻿3.8682 vs ﻿4.1254 |
| ^30^ | ﻿Ravichandran (2024) | MIMIC-III | 10,210 | S  U | Intermediate  (No) | S: LSTM, Liner layers U: ﻿ClinicalBERT | ﻿Fully connected layer | ﻿In-hospital mortality | Randomly split | ﻿Attention, Integrated gradients | 0.83 vs 0.80 |
| ^31^ | ﻿An (2024) | MIMIC-III; MIMIC-IV | ﻿MIMIC-III: 16,143  MIMIC-IV: ﻿19,242 | S  U | Intermediate | S: ﻿Bi-LSTM U: ﻿Word2Vec + CNN | ﻿Fully connected layer | ﻿In-hospital mortality | Randomly split | NA | MIMIC-III: 0.899 vs 0.871 ﻿MIMIC-IV: 0.890 vs 0.872 |
| ^32^ | Wang (2024) | MIMIC-IV; ﻿  MIMIC-CXR | ﻿11,636 | S  U  I: Chest X-ray | Intermediate  (No) | S: ﻿Bidirectional LSTM  U: ﻿Doc2Vec + LSTM I: ﻿ResNet | ﻿Fully connected layer | 1: ﻿In-hospital mortality 2: Long length of stay prediction 3: Readmission prediction | Randomly split | SHAP | 1: ﻿0.825 vs ﻿0.763 2: ﻿0.735 vs ﻿0.704 3: ﻿0.629 vs ﻿0.551 |
| ^33^ | Zhou (2024) | Development: Shanghai Jiao Tong University School of Medicine Affiliated Renji Hospital  External 1: Shanghai Public Health Clinical Center  External 2: Shanghai Jiao Tong University Affiliated Sixth People’s Hospital | Develop: 350 Internal: 150 Prospective: 181 External 1: 155 External 2: 92 | S  I: CT | Early  (Yes) | S: Raw data I: Handcrafted features | ﻿XGB | ﻿Acute respiratory distress syndrome prediction | Randomly split | SHAP | ﻿Internal: 0.916 vs 0.860 External 1: 0.865 (no comparison) Externa 2: 0.901 (no comparison) Prospective: 0.876 (no comparison) |
| ^34^ | ﻿Cui (2024) | MIMIC-III | ﻿12,289 | S  U | NA  (Yes) | ﻿Modality Specific Search | ﻿Fully connected layer | ﻿(1) Acute respiratory failure  (2) Shock  (3) Mortality (4) Diagnoses | Randomly split | NA | 1: ﻿0.7565 vs ﻿0.7377 2: ﻿0.7463 vs ﻿0.7364 3: ﻿0.8900 vs ﻿0.8876 4: ﻿R@30 0.4780 vs 0.2501 |
| ^35^ | ﻿Lee (2023) | MIMIC-IV | 30,954 | S  U | Intermediate  (No) | S: Transformer U: Transformer | MLP | ﻿1: Mortality 2: Vasopressor need prediction | K-fold cross validation | Integrated gradients | 1: 0.889 vs 0.852 2: 0.851 vs 0.805 |
| ^36^ | ﻿Khader (2023) | MIMIC-IV; ﻿  MIMIC-CXR | ﻿6,125 | S  I: Chest X-ray | Intermediate  (Yes) | S: ﻿Linear layer  I: VIT | MLP | ﻿In-hospital mortality | Randomly split | NA | ﻿0.863 vs 0.811 |
| ^37^ | ﻿Khader (2023) | MIMIC-IV; ﻿MIMIC-CXR Local: University Hospital Aachen, Aachen, Germany | ﻿﻿36,542 Local: 45,016 (not external) | S  I: Chest X-ray | Intermediate  (Yes) | S: ﻿﻿Perceiver I: VIT | MLP | MIMIC: predict 25 pathologic conditions Local: ﻿predict pleural effusion, pulmonary opacities, pulmonary congestion, and cardiomegaly | Randomly split | Attention | MIMIC: 0.77 vs 0.72 Local: 0.84 vs 0.83 |
| ^38^ | ﻿King (2023) | ﻿MIMIC-III | 75,165 | S  U | Intermediate  (Yes) | S: ﻿Linear layer + transformer U: ﻿Multi-layer transformer | ﻿Fully connected layer | ﻿1: In-hospital mortality  2: Phenotyping tasks | Randomly split | NA | 1: ﻿0.856 vs ﻿0.855 2: ﻿0.742 vs ﻿0.770 |
| ^39^ | ﻿Jaotombo (2023) | MIMIC-III | ﻿30,764 | S  U | Early  (Yes) | S: ﻿Handcrafted features U: ﻿﻿Bag of Words ﻿followed by Latent Dirichlet Allocation (LDA) dimension reduction | ﻿AutoGluon (14 classifiers) | ﻿Hospital length of stay | K-fold cross validation | Permutation feature importance, LIME | 0.963 vs 0.944 |
| ^40^ | ﻿Zhang (2023) | MIMIC-III | 16,722 | S  U | Intermediate  (No) | S: ﻿ConvTransformer U: ﻿BioBERT | ﻿Fully connected layer | ﻿In-hospital mortality | Randomly split | NA | 0.8894 vs 0.8553 |
| ^41^ | ﻿Zhang (2023) | MIMIC-III | 22,350 | S  U | Intermediate  (Yes) | S: CNN + ﻿multi-time attention + gating U: ﻿TextEncoder + multi-time attention | ﻿Fully connected layer | ﻿1: 48-hour in-hospital mortality 2: 24-hour phenotype classification | Randomly split | NA | 1: F1-score 56.45 vs 52.57 2: 86.06 vs 85.43 |
| ^42^ | Lee (2023) | MIMIC-III | ﻿30,060 | S  U | Intermediate  (Yes) | S: ﻿Bidirectional GRU U: Clinical BioBERT + Transformer | MLP | 1: ﻿In-hospital mortality ﻿2: In-ICU mortality ﻿3: LOS > 3 d ﻿4: LOS > 7 d | Randomly split | Attention | ﻿1: 0.9005 vs 0.8763 ﻿2: ﻿0.9048 vs 0.8865 ﻿3: ﻿0.7835 vs 0.7697 ﻿4: 0.8513 vs 0.8314 |
| ^43^ | Niu (2023) | MIMIC-III | 16,722 | S  U | Intermediate  (No) | S: LSTM U: ﻿BioBERT | ﻿Fully connected layer | ﻿In-hospital mortality | Randomly split | NA | 0.8835 vs ﻿﻿0.8553 |
| ^44^ | Xu (2023) | MIMIC-III | 45,152 | S  U | Intermediate  (Yes) | S: ﻿Self-supervised Transformer for Time-Series U: ﻿﻿clinical BERT | ﻿Fully connected layer | ﻿1. Forecast physiological feature values in the two hours 2. Sepsis prediction | Randomly split | NA | 1. MSE: ﻿5.2493 vs ﻿5.2455 2. 0.889 vs ﻿0.891 |
| ^45^ | ﻿Kyung (2023) | MIMIC-III; MIMIC-III Waveform Database | 510 | S  W: ﻿PPG, ECG | Early + Intermediate  (No) | S: Raw data W: ﻿GRU | ﻿Fully connected layer | ﻿Blood pressure estimation | Randomly split | NA | SBP: MAE 4.24 vs 5.11 DBP: MAE 3.01 vs 3.34 |
| ^46^ | Wang (2023) | I-CARE | 607 | S W: EEG | Late  (No) | S: XGBoost W: CNN + RNN | ﻿Weighted sum | Neurological outcomes (Good and poor) | NA | NA | 0.77 vs 0.66 |
| ^47^ | ﻿Krones (2023) | I-CARE | 607 | S  W: EEG, ECG, SpO2 | Early + Intermediate + Late  (Yes) | S: Raw data W: Handcrafted features + DenseNet + Ridge regression classifier | RF | ﻿Neurological outcomes (Good and poor) | K-fold cross validation | NA | ﻿0.854 (no comparison) |
| ^48^ | ﻿Amiri (2023) | Rigshospitalet, Copenhagen University Hospital | 48 | W: ﻿fMRI I: EEG | Early  (Yes) | W: Handcrafted features I: Handcrafted features | SVM or RF | ﻿Predict residual consciousness in acute disorders of consciousness patients (binary) 1: At ﻿study enrolment 2: At ICU discharge | K-fold cross validation | NA | Primary: 0.78 vs 0.78 Second: 0.83 vs 0.81 |
| ^49^ | Lin (2023) | MIMIC-III | 33,678 | S  U | Intermediate  (No) | S: ﻿ON-LSTM + multi-head self-attention layer U: ﻿Multi-channel convolution module | ﻿Fully connected layer | 1: In-hospital mortality  2: decompensation prediction | Randomly split | Attention | 1: ﻿0.9130 vs 0.8787 2: ﻿0.9661 vs ﻿0.9410 |
| ^50^ | ﻿Chiu (2023) | MIMIC-III | ﻿ 27,550 | S  U | Early  (No) | S: Raw data U: ﻿Latent Dirichlet Allocation | GBM | ﻿1. Mortality at 3 days 2. Mortality at 30 days  3. Mortality at 365 days | K-fold cross validation | Tree-based feature ranking | 1: ﻿0.8820 vs ﻿0.8598 2: ﻿0.7815 vs ﻿0.7634  3: ﻿0.7754 vs ﻿0.7588 |
| ^51^ | Wang (2022) | MIMIC-III | ﻿32,310 | S  U | Intermediate  (No) | S: Bidirectional LSTM U: ﻿﻿GloVe + ﻿Bidirectional LSTM | MLP | ﻿ICU patient mortality prediction | NA | NA | Accuracy: 90.43 (no comparison) |
| ^52^ | ﻿Duvieusart (2022) | MIMIC-IV; ﻿  MIMIC-CXR | ﻿2774 | S  I: Chest X-ray | Early  (No) | S: Handcrafted features I: Handcrafted features | ﻿XGB | ﻿Cardiomegaly classification: positive, negative | K-fold cross validation | NA | 0.813 vs 0.810 |
| ^53^ | ﻿Salekin (2022) | University of South Florida Multimodal Neonatal Pain Assessment Dataset | 45 | W: Audio (crying and background noises  V: Video (face and body) | Intermediate  (No) | W: Google’s VGGish + LSTM-based AE V: FaceNet-based, Resnet18-based + LSTM-based AE | MLP | 1. Pain classification 2. Intensity estimation (﻿ranges from 0 to 7) | ﻿Leave-one-out cross validation | NA | 1: 0.906 vs ﻿0.835 2: ﻿MAE 1.73 (no comparison) |
| ^54^ | ﻿Silva (2022) | MIMIC-III | ﻿34,560 | S  U | Intermediate  (Yes) | S: Dense layer  U: Clinical BERT, SapBERT + Dense layer | ﻿Fully connected layer | ﻿1: Unexpected patient readmission  2: ﻿Diagnoses prediction | K-fold cross validation | NA | 1: ﻿0.871 vs 0.797 2: ﻿AUPRC: 0.444 vs 0.437 |
| ^55^ | ﻿Ahuja (2022) | MIMIC-III | 38,597 | S  U | Intermediate  (No) | S: Handcrafted features U: Handcrafted features | Bayesian inference | ﻿Automatic phenotyping | NA | Probabilistic model | NA |
| ^56^ | ﻿Jabbour (2022) | Development and internal: ﻿an academic medical center in the upper Midwest (Michigan Medicine) External: MIMIC-IV; ﻿  MIMIC-CXR | ﻿Develop: 1618 External: 1774 | S  I: Chest X-ray | NA  (Yes) | S: Raw or MLP I: ﻿ Pretrained DenseNet-121 (frozen) | ﻿Fully connected layer | ﻿Pneumonia, heart failure, chronic obstructive pulmonary disease prediction | Randomly split | Grad-CAM | Internal: 0.82 vs 0.78 External: ﻿0.78 vs 0.75 |
| ^57^ | ﻿Cheng (2022) | University of Pennsylvania Health System in Philadelphia External: Brown University-affiliated hospitals in Providence | Develop: 546 External: 108 | S  I: Chest X-ray | Late  (Yes) | S: MLP I: Resnet-50 + ViT + MLP | Weighted sum | In-hospital mortality of COVID-19 patients | Randomly split | NA | Not report internal results External: 0.727 vs 0.702 |
| ^58^ | ﻿Hayat (2022) | MIMIC-IV; ﻿  MIMIC-CXR | 11,215 | S  I: Chest X-ray | Intermediate  (Yes) | S: Two stacked layers of an LSTM I: ﻿ResNet-34 | ﻿Fully connected layer | ﻿1. Phenotype classification 2. ﻿In-hospital mortality prediction | Randomly split | NA | 1: ﻿0.770 vs ﻿0.746 2: ﻿0.865 vs ﻿0.833 |
| ^59^ | ﻿Soenksen (2022) | MIMIC-IV; ﻿  MIMIC-CXR | 6485 | S  U  I: Chest X-ray | Early  (Yes) | S: Handcrafted features U: Clinical BERT I: Densenet121-res224-chex | ﻿XGB | 1. 10 distinct chest pathology diagnoses 2. Length-of-stay  3. 48 h mortality predictions | Randomly split | SHAP | 1. ΔAUROC: 6-22% 2. 0.939 vs 0.919 3. 0.912 vs 0.889 |
| ^60^ | ﻿ Zhao (2022) | MIMIC-III | ﻿ 8577 | S  U | Intermediate  (No) | S: Liner layer, LSTM U: ClinicalBERT | MLP | ﻿1: Predict shock 2: Acute respiratory failure at 12h 3: In-hospital mortality at 48h  4: Diagnoses at 48h | Randomly split | NA | 1: ﻿0.836 vs ﻿﻿0.802 2: ﻿0.781 vs ﻿0.756 3: ﻿0.892 vs ﻿0.857 4: ﻿Recall@10: ﻿0.385 vs 0.336 |
| ^61^ | ﻿Mollura (2021) | MIMIC-III; MIMIC-III Waveform Database | 142 | S  W: ECG, ABP | Early  (Yes) | S: Raw data W: Handcrafted features | SVM | Sepsis prediction | Randomly split | Partial dependence plots | 0.92 (no comparison) |
| ^62^ | ﻿Grant (2021) | MIMIC-IV; ﻿  MIMIC-CXR | 2404 | S  I: Chest X-ray | Intermediate  (No) | S: MLP I: ResNet-50 | MLP | ﻿Classification of Cardiomegaly (positive, negative) | K-fold cross validation | Grad-CAM | 0.880 vs 0.840 |
| ^63^ | Yang (2021) | MIMIC-III | 14,698 | S  U | Intermediate  (No) | S: LSTM U: Label-aware ﻿attention + CNN | ﻿Fully connected layer | ﻿In-hospital mortality | NA | Attention | 0.861 vs 0.844 |
| ^64^ | ﻿Harerimana (2021) | MIMIC-III | ﻿47,796 | S  U | Intermediate  (No) | S: Raw data then encode U: ﻿﻿Cui2Vec and ClinicalBERT + Bi-GRU | ﻿Fully connected layer | ﻿1: In-hospital mortality ﻿2: Length of stay (3 classes) | Randomly split | Attention | 1: 0.872﻿ (no comparison) 2: 0.821 (no comparison) |
| ^65^ | Lin (2021) | MIMIC-IV;  ﻿MIMIC-CXR | ﻿9,928 | S  U  I: Chest X-ray | Intermediate  (Yes) | S: MLP U: ﻿BlueBERT + MLP I: CNN + MLP | MLP + Cox | ﻿ICU mortality | Randomly split | NA | ﻿C-index: ﻿0.7847 vs ﻿0.7733 |
| ^66^ | ﻿Shickel (2021) | ﻿University of Florida Health | 51 | S  W: activity | Intermediate  (No) | S: GRU W: GRU | ﻿Fully connected layer | ﻿Patient illness severity (successful vs unsuccessful hospital discharge) | K-fold cross validation | NA | ﻿0.915 vs ﻿0.828 |
| ^67^ | ﻿Salekin (2021) | University of South Florida Multimodal Neonatal Pain Assessment Dataset | 45 | W: Audio (crying and background noises  V: Video (face and body) | Late  (No) | V: Face (Bilinear CNN + LSTM), body (VGG-16 + MLP + LSTM) W: VGG-16 + MLP | Unweighted majority voting | ﻿Neonatal postoperative pain assessment | ﻿Leave-one-out cross validation | NA | 0.901 vs 0.869 |
| ^68^ | An (2021) | MIMIC-III | ﻿3844 | S  U | Intermediate  (No) | S: Linear layer U: Self-attention + Transformer; Word2vec + Text-CNN | ﻿Fully connected layer | ﻿Diagnosis prediction | K-fold cross validation | NA | Recall@20: ﻿0.6536 vs ﻿0.6383 Precision@20: ﻿0.4017 vs 0.3923 |
| ^69^ | Xu (2021) | MIMIC-III | 40,511 | S  U | Intermediate + Late  (No) | S: Transformer U: Transformer | ﻿Fully connected layer | 1. Predict the primary Clinical Classifications Software (CCS) diagnosis code 2. Predict the International Classification of Diseases | Randomly split | NA | 1. Recall@5: 0.9075 vs 0.8841 2. AUPRC: 0.3327 vs 0.3200 |
| ^70^ | ﻿Yang (2021) | MIMIC-III | ﻿14,174 | S  U | Intermediate  (Yes) | S: Linear, LSTM U: ﻿ClinicalBERT | ﻿Fully connected layer | 1. ﻿Predict the diagnoses 2. ﻿Acute respiratory failure | Randomly split | NA | 1: ﻿0.792 vs ﻿0.772 2: Recall @20 ﻿0.490 vs ﻿﻿0.479 |
| ^71^ | ﻿Deznabi (2021) | MIMIC-III | 16,722 | S  U | Intermediate  (Yes) | S: LSTM U: BioClinical BERT | ﻿Fully connected layer | ﻿In-hospital mortality | Randomly split | NA | ﻿0.899 vs ﻿0.875 |
| ^72^ | ﻿Hayat (2021) | MIMIC-IV; ﻿  MIMIC-CXR | 11,215 | S  I: Chest X-ray | Intermediate  (No) | S: LSTM I: ﻿ ResNet-34 | ﻿Fully connected layer | ﻿25 phenotype prediction | Randomly split | NA | ﻿0.764 vs ﻿0.740 |
| ^73^ | ﻿Bardak (2021) | MIMIC-III | ﻿21,080 | S  U | Intermediate  (Yes) | S: GRU U: ﻿med7 (﻿Named Entity Recognition model) + word embedding+1D CNN | ﻿Fully connected layer | 1: In-hospital mortality 2: In-ICU mortality 3: LOS > 3  4: LOS > 7 | Randomly split | NA | 1: ﻿87.55 vs ﻿85.04 2: ﻿88.35 vs ﻿86.32 3: ﻿69.93 vs ﻿67.40  4: ﻿72.55 vs ﻿70.54 |
| ^74^ | ﻿Hong (2020) | Children Healthcare of Atlanta | 57 | S  W: ECG | Late  (Yes) | S: ﻿Random forest, LR W: ﻿ResNeXt | ﻿Ensemble individual scores | ﻿Postsurgical patient is getting stable or stay still critical (binary) | Temporal split | NA | ﻿0.9551 (no comparison) |
| ^75^ | ﻿Darabi (2020) | MIMIC-III | ﻿38,597 | S  U | Intermediate  (Yes) | S: Raw data; Transformer encoder U: ﻿BioBERT followed by a bidirectional GRU | MLP | 1. ﻿30-day unplanned readmission to the ICU 2. ICU mortality 3. Long length of stay 4. Code prediction task | K-fold cross validation | NA | 1. ﻿67.42 vs ﻿﻿64.53 2. ﻿63.42 vs ﻿60.41 3. ﻿Top-1 25.57 vs ﻿﻿22.22 4. ﻿Diagnosis@20 ﻿65.11  Procedure@20 ﻿62.25 (no comparison) |
| ^76^ | ﻿Hammoud (2020) | ﻿MIMIC-II | ﻿17,763 | S  U | ﻿Early + Intermediate  (No) | S: Raw data U: ﻿Bag-of-words + Transformer | ﻿Lasso regression | ﻿Early septic shock prediction | K-fold cross validation | NA | ﻿0.8899 (no comparison) |
| ^77^ | Zhang (2020) | MIMIC-III | 39,429 | S  U | Intermediate  (Yes) | S: CNN or LSTM U: ﻿Doc2Vec + CNN or BiLSTM | ﻿Fully connected layer | 1: In-hospital mortality 2: 30-day hospital readmission  3: Long length of stay prediction | Randomly split | NA | 1: 0.871 vs 0.837 2: 0.674 vs 0.663 3: 0.784 vs 0.757 |
| ^78^ | ﻿Khadanga (2019) | MIMIC-III | 22,353 | S  U | Intermediate  (Yes) | S: LSTM U: ﻿Pre-trained word2vec embeddings + CNN | ﻿Fully connected layer | ﻿1. In-hospital Mortality 2. ﻿Decompensation: ﻿whether the person dies in ICU within the next 24 hours 3. ﻿Length of Stay Forecasting | Randomly split | NA | 1. ﻿0.865 vs ﻿0.844 2. ﻿0.907 vs ﻿0.892 3. ﻿Kappa ﻿0.453 vs ﻿0.438 |
| ^79^ | Xu (2019) | MIMIC-III | ﻿44,659 | S  U | Late  (No) | S: Decision tree U: Text-CNN and Char-CNN + Bid LSTM | ﻿Weighted sum | ﻿Predict ICD-10 diagnostic codes | Randomly split | Path-based attribution, LIME | 0.954 vs 0.931 |
| ^80^ | ﻿Feng (2019) | MIMIC-III; MIMIC-III Waveform Database | ﻿6177 | S  W: ECG | Intermediate  (Yes) | S: LSTM W: CNN-LSTM | ﻿Fully connected layer | 1st 24h data to predict mortality in next 24h | K-fold cross validation | NA | ﻿0.9195 (no comparision) |
| ^81^ | ﻿Jin (2018) | MIMIC-III | ﻿ 33,798 | S  U | Intermediate  (No) | S: LSTM U: Entity recognition + dense layer | ﻿Fully connected layer | In-hospital mortality | Randomly split | NA | ﻿0.8734 vs ﻿0.8531 |
| ^82^ | Xu (2018) | MIMIC-III; MIMIC-III Waveform Database | ﻿6670 | S  W: ECG | Intermediate  (No) | S: Handcrafted features W: CNN | LSTM | ﻿1. Mortality next 24 hours 2. ﻿Length of stay | Randomly split | Attention | 1. ﻿90.18 vs ﻿88.19 2. ﻿Accuracy ﻿86.82 vs ﻿85.34 |
| ^83^ | Sen (2017) | MIMIC-III | 1328 | S  U | Late  (No) | S: Handcrafted features U: Text mining (Meta learner: SVM, RF, LR) | SVM | Prediction for Clostridium Diﬃcile Infection | Randomly split | NA | 0.838 vs 0.832 |
| ^84^ | Huddar (2016) | MIMIC-II | ﻿ 775 | S  U | Early  (No) | S: Handcrafted features U: ﻿Extract term frequencies of statistically significant terms within each heading, after clustering the headings | LR | ﻿Postoperative respiratory failure prediction | K-fold cross validation | NA | 0.881 vs 0.873 |
| ^85^ | ﻿Ghassemi (2014) | MIMIC-II | ﻿19,308 | S  U | Early  (No) | S: Raw data U: ﻿Latent Dirichlet Allocation | SVM | 1: In-hospital mortality 2: 30 day post-discharge mortality 3: 1 year post-discharge mortality | Randomly split | ﻿Enrichment measures | 1: ﻿0.961 vs 0.944 2: ﻿0.818 vs ﻿0.783 3: ﻿0.813 vs ﻿0.776 |
| ^86^ | ﻿Saria (2010) | ﻿Stanford Lucile Packard Children Hospital | ﻿275 | S  U | Early  (No) | S: Handcrafted features U: Handcrafted features | LR | ﻿Automatic coding of patient outcomes | Randomly split | NA | F1 score: ﻿88.3 vs ﻿84.7 |

^a^ S = Structured Data, I = Imaging, W = Waveforms, U = Unstructured Text, V = Video.

^b^ Unless a specific metric is indicated in the table, the default metric is AUC.

Appendix Table 3. Single-modality feature-extraction methods prior to fusion

| **Modality** | **Data** | **Method type (count ^a^)** | **Specific methods (count ^a^)** |
| --- | --- | --- | --- |
| Structured Data | ﻿Demographics, vital signs, laboratory test, medication records, clinical scores, etc. | RNN-related (25) | LSTM (16), Bi-LSTM (3), GRU (3), Bi-GRU (1), ﻿Ordered neuron-LSTM (1), RNN (1) |
|  |  | Raw data (12) | |
|  |  | Handcrafted features (12) | |
|  |  | Simple neural network (17) | Linear layer (10), MLP (7) |
|  |  | CNN (9) | |
|  |  | Transformer-related (8) | Transformer (4), TabTransformer (1), T-transformer (1), ConvTransformer (1), Self-supervised Transformer for Time-Series (1) |
|  |  | Traditional model (6) | XGBoost (2), Gradient boosting machine (1), Random Forest (1), Logistic regression (1), Decision tree (1) |
|  |  | Others (9) | GCN (2), Encoder (2), Time2Vec (2), Piecewise linear encoding (1), HIVE-COTE (1), Perceiver (1) |
| Imaging | ﻿Chest X-ray, ﻿Abdominal X-ray, CT, MRI, Ultrasound | ResNet-related (8) | ResNet-50 (3), ResNet-34 (3), ResNet (2) |
|  |  | Handcrafted features (6) | |
|  |  | CNN (4) | |
|  |  | DenseNet-related (4) | DenseNet121 (1), DenseNet121-Res224-CheX (2), Pretrained DenseNet-121 (1) |
|  |  | Others (8) | Vision transformer (3), MobileNet (1), ChexNet (1), Swin-transformer (1), LSTM (1), InceptionResnet-V2 (1) |
| Waveforms | EEG, ECG, PPG, Arterial blood pressure, Respiratory sounds, Activity, Audio | Handcrafted features (6) | |
|  |  | RNN-related (5) | GRU (2), LSTM (2), RNN (1) |
|  |  | Others (12) | CNN (3), MLP (2), Variational autoencoder (1), HIVE-COTE (1), DenseNet (1), VGG-16 (1), ResNeXt (1), Google’s VGGish (1), Ridge regression classifier (1) |
| Unstructured Text | Radiology reports, Diagnosis reports, Electrocardiogram notes, Procedure notes, Chief complaint, Nursing documentation, ﻿Discharge summaries, etc. | BERT-series (23) | ClinicalBERT (9), BioBERT (5), BioClinicalBERT (4), BlueBERT (2), SapBERT (1), Clinical BioBERT (1), BioMedBERT (1) |
|  |  | CNN (11) | CNN (7), Text-CNN (1), Char-CNN (1), IMUI-CNN (1), GloVec-CNN (1) |
|  |  | Static Embedding (8) | Word2Vec (3), Doc2Vec (2), Cui2Vec (1), BioSent2Vec (1), GloVec (1) |
|  |  | Transformer-related (7) | Transformer (6), Longformer (1) |
|  |  | RNN-related (7) | Bi-LSTM (3), LSTM (2), Bi-GRU (2) |
|  |  | Text Mining (5) | Named Entity Recognition (1), Entity recognition (1), Knowledge extraction system (1), Apache clinical text analysis (1), Text mining (1) |
|  |  | Handcrafted features (3) | |
|  |  | Large language model (2) | Google-T5 (1), Qwen-1.5 32B (1) |
|  |  | Others (7) | Latent dirichlet allocation (3), TextEncoder (2), Bag-of-words (1), GCN (1) |
| Video | Recordings from incubator neonate monitoring camera | Autoencoder (3) | FaceNet-based autoencoder (1), Resnet18-based autoencoder (1), LSTM-based autoencoder (1) |
|  |  | Others (4) | Bilinear CNN (1), VGG-16 (1), LSTM (1), MLP (1) |

**^a^** Refers to the total frequency of occurrence across all included studies, where multiple methods may be reported within a single study. Bi = Bidirectional, BERT = Bidirectional Encoder Representations from Transformers, CNN = Convolutional Neural Network, EEG = Electroencephalogram, ECG = Electrocardiogram, GCN: Graph Convolutional Network, GRU = Gated Recurrent Unit, HIVE-COTE = Hierarchical Vote Collective of Transformation-based Ensembles, LSTM = Long Short-Term Memory, MLP = Multilayer perceptron, MRI = Magnetic Resonance Imaging, PPG = Photoplethysmogram, RNN = Recurrent Neural Network, VGG = Visual Geometry Group, Vec = Vector

Appendix Table 4. Models applied in the presence of missing modalities

| **Reference** | **Author (Year)** | **Modality used ^a^** | **Fusion Method** | **Methods for dealing with missing modalities** |
| --- | --- | --- | --- | --- |
| ^9^ | Guo (2025) | S + I | Intermediate | Uncertainty-Aware dynamic weighting |
| ^36^ | ﻿Khader (2023) | S + I | Intermediate | Set to zero in Transformer |
| ^37^ | ﻿Khader (2023) | S + I | Intermediate | Set to zero in Transformer |
| ^53^ | ﻿Salekin (2022) | I + V | Intermediate | Generative model that combines all the modalities while learning to reconstruct any missing modalities |
| ^58^ | ﻿Hayat (2022) | S + I | Intermediate | LSTM to handle input sequences of variable length, in case of a missing modality |
| ^72^ | ﻿Hayat (2021) | S + I | Intermediate | ﻿A novel dynamic approach towards integrating auxiliary data modalities﻿, it inherently handles missingness |

^a^ S = Structured Data, I = Imaging, V = Video.

LSTM = Long Short-Term Memory

Appendix Table 5. Missing values imputation methods for single modalities

| **Reference** | **Author (Year)** | **Modality used** | **Missing data imputation methods** |
| --- | --- | --- | --- |
| ^3^ | ﻿Mendoza (2025) | S + W | W: Forward fill; specific value imputation; mean imputation |
| ^7^ | Slobbe (2025) | S + U | S: Forward fill; mean imputation |
| ^18^ | Liu (2024) | S + U | S: Forward fill; mean imputation |
| ^14^ | Wang (2024) | S + U | S: Forward fill |
| ^17^ | Gao (2024) | S + U | S: Median imputation |
| ^18^ | Lin (2024) | S + I | S: Predictive mean matching algorithm |
| ^19^ | Tan (2024) | S + U | S: Forward fill |
| ^23^ | ﻿Saleh (2024) | S + U | S: Mean imputation |
| ^24^ | ﻿Insalata (2024) | S + U | S: Forward fill |
| ^25^ | ﻿Ayden (2024) | S + U | S: Forward fill; backward fill; zero imputation |
| ^29^ | Chen (2024) | S + I + U | S: Zero imputation |
| ^30^ | ﻿Ravichandran (2024) | S + U | S: Forward fill; specific value imputation |
| ^31^ | ﻿An (2024) | S + U | S: Forward fill; specific value imputation |
| ^33^ | Zhou (2024) | S + I | S: MICE |
| ^34^ | ﻿Cui (2024) | S + U | S: Forward fill |
| ^35^ | ﻿Lee (2023) | S + U | S: Forward fill; zero imputation |
| ^36^ | ﻿Khader (2023) | S + I | S: Forward fill; specific value imputation |
| ^38^ | ﻿King (2023) | S + U | S: Forward fill; specific value imputation |
| ^39^ | ﻿Jaotombo (2023) | S + U | S: Linear interpolation |
| ^40^ | ﻿Zhang (2023) | S + U | S: GAN |
| ^41^ | ﻿Zhang (2023) | S + U | S: Forward fill; mean imputation |
| ^43^ | Niu (2023) | S + U | S: Forward fill; specific value imputation |
| ^44^ | Xu (2023) | S + U | S: Forward fill |
| ^49^ | Lin (2023) | S + U | S: Forward fill; specific value imputation |
| ^59^ | ﻿Soenksen (2022) | S + I + U | S: Zero imputation |
| ^60^ | ﻿ Zhao (2022) | S + U | S: Forward fill |
| ^66^ | ﻿Shickel (2021) | S + W | S: Forward fill; median imputation |
| ^67^ | ﻿Salekin (2021) | W + V | V: Resampling |
| ^69^ | Xu (2021) | S + U | S: Forward fill |
| ^71^ | ﻿Deznabi (2021) | S + U | S: Forward fill; specific value imputation |
| ^76^ | ﻿Hammoud (2020) | S + U | S: Forward fill; mean imputation |
| ^77^ | Zhang (2020) | S + U | S: Zero imputation |
| ^78^ | ﻿Khadanga (2019) | S + U | S: Forward fill; specific value imputation |
| ^79^ | Xu (2019) | S + U | S: Zero imputation |
| ^80^ | ﻿Feng (2019) | S + W | S: Forward fill |
| ^81^ | ﻿Jin (2018) | S + U | S: Forward fill; specific value imputation |
| ^82^ | Xu (2018) | S + W | S: Forward fill |
| ^83^ | Sen (2017) | S + U | S: Mean imputation |

S = Structured Data, I = Imaging, W = Waveforms, U = Unstructured Text, V = Video, MICE = Multiple Imputation by Chained Equations, GAN = Generative Adversarial Network.

Appendix Table 6. Performance comparison of the multimodal model versus clinician

AUC = Area Under the Curve, XAI = Explainable artificial intelligence, ICD = International Classification of Diseases.

| **Reference** | **Author (Year)** | **Clinical task** | **Comparison between multimodal model with clinicians** |
| --- | --- | --- | --- |
| ^11^ | Liu (2025) | Medical report generation | Model-generated reports were preferred or considered equivalent to clinician reports in 53.8% of cases, indicating substantial agreement |
| ^26^ | Cui (2024) | Early diagnosis of necrotizing enterocolitis in newborns | In prospective validation of 50 pediatric cases, the model achieved an AUC similar to clinicians (0.83 vs. 0.82), reflecting comparable diagnostic performance |
| ^30^ | Ravichandran (2024) | In-hospital mortality prediction | Comparative analysis of 200 cases showed that multimodal systems with XAI extract clinically relevant information at a level similar to physicians |
| ^56^ | Jabbour (2022) | Pneumonia, heart failure, chronic obstructive pulmonary disease prediction | The model demonstrated equal or superior performance to randomly selected physicians (AUC 0.84 vs. 0.79), supporting its utility as a decision aid. |
| ^79^ | Xu (2019) | Predict ICD-10 diagnostic codes | ﻿The model was evaluated on a test set of 25 samples by comparing the overlap scores between physicians’ annotations and the outputs of the model. For the text-oriented evaluation, the model achieved an average Jaccard Similarity Coefficient (JSC) of 0.1806. In the table-oriented evaluation, the average JSC between the model and the physicians’ annotations was 0.3105. The model could identify a wider range of diagnostic features than clinicians, offering additional clinically relevant insights. |

Appendix Table 7. Guidelines reported in studies for model development

| **Reference** | **Author** | **Clinical task** | **Guidelines** |
| --- | --- | --- | --- |
| ^17^ | Gao (2024) | Hospital mortality prediction for ﻿heart failure patients | TRIPOD reporting guidelines |
| ^33^ | Zhou (2024) | ﻿Acute respiratory distress syndrome prediction | TRIPOD-AI guidelines |
| ^56^ | ﻿Jabbour (2022) | ﻿Pneumonia, heart failure, chronic obstructive pulmonary disease prediction | TRIPOD reporting guidelines |

TRIPOD = Transparent Reporting of a multivariable prediction model for Individual Prognosis Or Diagnosis, AI = Artificial intelligence.

Appendix Table 8. Manually searched additional open-source multimodal ICU databases from websites or PhysioNet

| **First released year** | **Database** | **Data source** | **ICU Patient type** | **No. of patients** | **Modality type ^a^** | **Link source** |
| --- | --- | --- | --- | --- | --- | --- |
| 2019 | Amsterdam University Medical Centers Database (AmsterdamUMCdb) | The department of Intensive Care, a mixed medical-surgical ICU, from Amsterdam University Medical Center, Amsterdam, The Netherlands (2003 - 2016) | Overall | 20,109 | S: Time-invariant and time-variant variables U: Free-text clinical notes | <https://amsterdammedicaldatascience.nl/> |
| 2019 | Paediatric-specific intensive care database (PIC) | The Children’s Hospital, Zhejiang University School of Medicine, Zhejiang, China (2010 - 2018) | Paediatrics | 12,881 | S: Time-invariant and time-variant variables U: Free-text clinical notes | <http://pic.nbscn.org/> |
| 2021 | Critical care database comprising patients with infection | ICUs in Zigong Fourth People’s Hospital, Sichuan, China (2019 - 2020) | ICU patients with infection | 2,790 | S: Time-invariant and time-variant variables U: Free-text clinical notes | <https://physionet.org/content/icu-infection-zigong-fourth/1.1/> |
| 2023 | Salzburg Intensive Care database (SICdb) | Department of Anesthesiology and Intensive Care Medicine at the General Hospital Salzburg and Paracelsus Medical University, Salzburg, Austria (2013 - 2021) | Overall | 21,583 | S: Time-invariant and time-variant variables U: Semi-structured notes (ICD10 main text, list of interventions, LOINC Code and name) | <https://www.sicdb.com/?home> |
| 2023 | Chinese critical care database in a tertiary care medical center | ICUs in Zhejiang Provincial People’s Hospital, Zhejiang, China (January 2012 - May 2022) | Overall | 7,638 | S: Time-invariant and time-variant variables U: Free-text clinical notes | <https://physionet.org/content/zhejiang-ehr-critical-care/1.0/> |
| 2024 | Northwestern ICU (NWICU) database | Northwestern Medicine (NM), a network of twelve hospitals located in Chicago and the surrounding area, US (2020 - 2022) | Overall | 23,204 | S: Time-invariant and time-variant variables U: Semi-structured notes (ICD10 main text, list of drugs) | <https://physionet.org/content/nwicu-northwestern-icu/0.1.0> |
| 2025 | ALarms, Outcomes Telemetry with Timing (ALOTT): a bedside-EMR database | The James Cancer Hospital and Ross Heart Hospital, Ohio, US (September 2018 - November 2020) | Not mentioned | 11,349 | S: Time-invariant and time-variant variables W: Electrocardiogram and blood oxygen U: Allergy reaction description in text and semi-structured notes | <https://physionet.org/content/alott/1.0.0/> |
|  | MIMIC-series ^b^ |  |  |  |  |  |
| 2022 | MIMIC-IV Waveform Database | Beth Israel Deaconess Medical Center in Boston, Massachusetts, US (link to MIMIC-IV) | Overall | 198 | W: ECG, ABP, respiration, PPG, etc. | <https://physionet.org/content/mimic4wdb/0.1.0/> |
| 2023 | MIMIC-IV-ECHO: Echocardiogram Matched Subset | Beth Israel Deaconess Medical Center in Boston, Massachusetts, US (link to MIMIC-IV, 2017 - 2019) | Overall | 4,579 | I: Echocardiogram | <https://physionet.org/content/mimic-iv-echo/0.1/> |
| 2023 | MIMIC-IV-ECG: Diagnostic Electrocardiogram Matched Subset | Beth Israel Deaconess Medical Center in Boston, Massachusetts, US (link to MIMIC-IV, 2008 - 2019) | Overall | ~160,000 | S: RR interval, QRS onset and end, etc. W: ECG U: Cardiologist reports | <https://physionet.org/content/mimic-iv-ecg/1.0/> |

^a^ S = Structured Data, I = Imaging, W = Waveforms, U = Unstructured Text.

^b^ The following databases are still part of the MIMIC series as mentioned in Table 2 of the main text, and therefore are not considered as new database names.

ABP = Arterial Blood Pressure, ECG = Electrocardiogram, ICU = Intensive Care Unit, ICD = International Classification of Diseases, LOINC = Logical Observation Identifier Names and Codes, MIMIC = Medical Information Mart for Intensive Care, PPG = Photoplethysmogram.


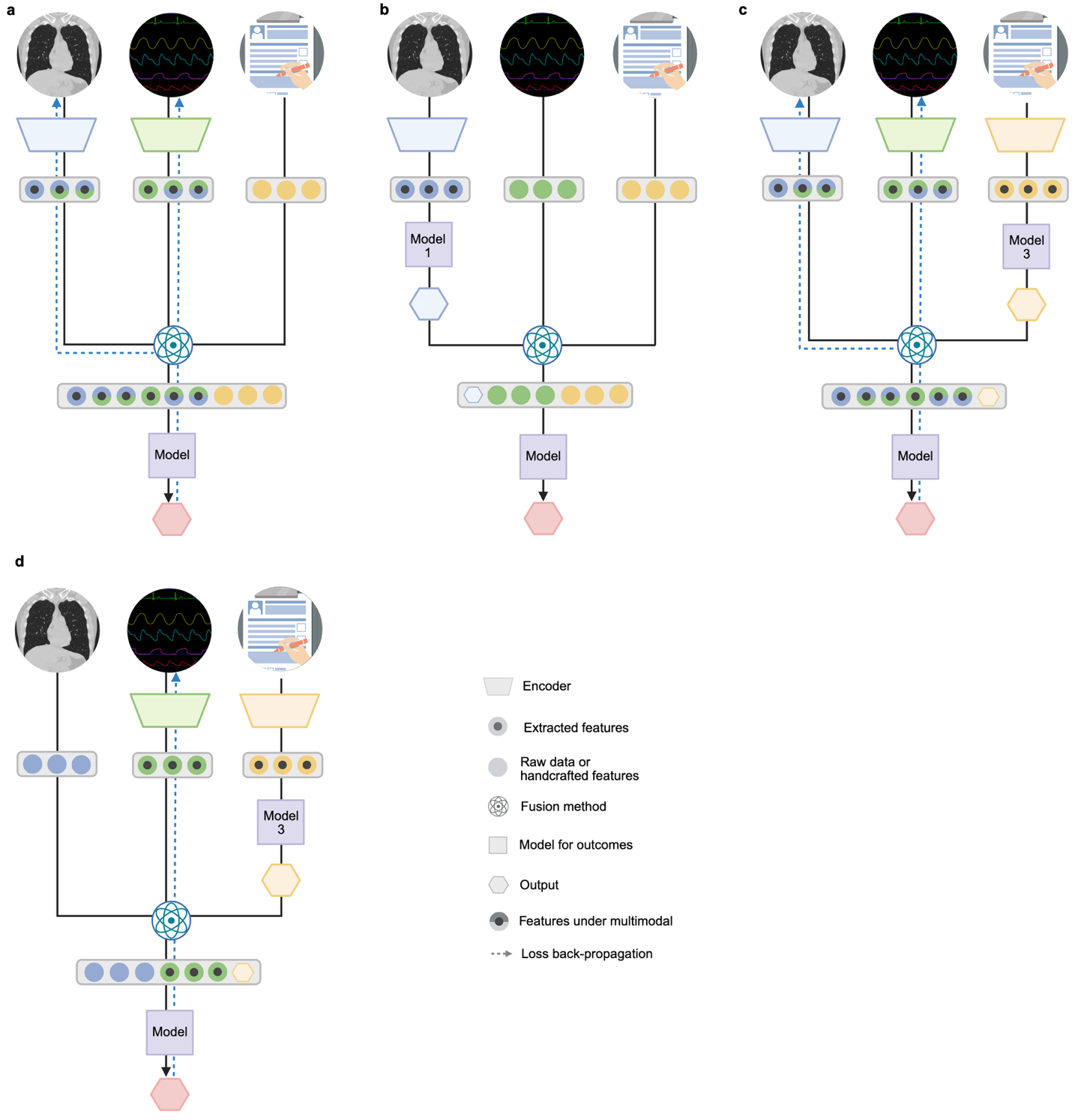


Appendix Figure 1. Mixed fusion strategies. a: Early + Intermediate fusion; b: Early + Late fusion; c: Intermediate + Late fusion; d: Early + Intermediate + Late fusion.


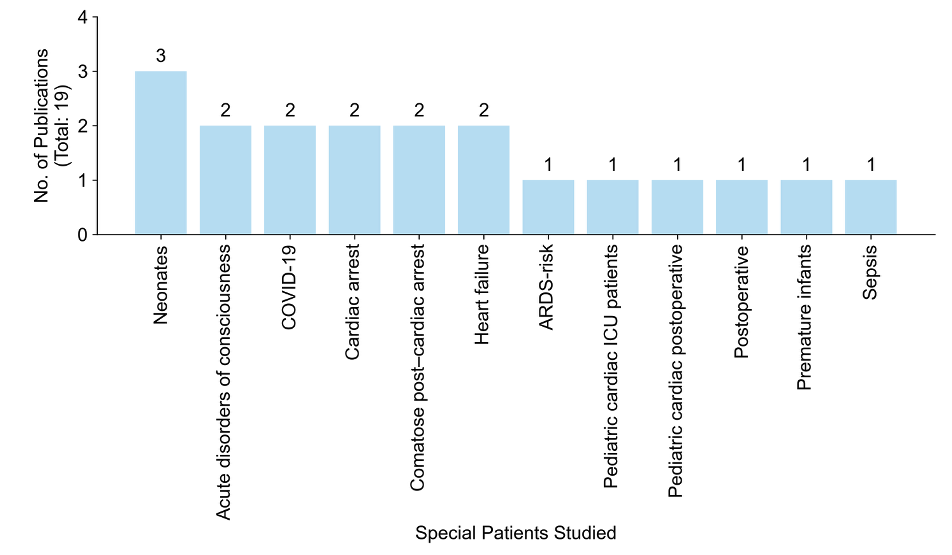


Appendix Figure 2. Specialized ICU populations targeted across included studies. ARDS = Acute Respiratory Distress Syndrome, COVID = Coronavirus Disease, ICU = Intensive Care Unit.


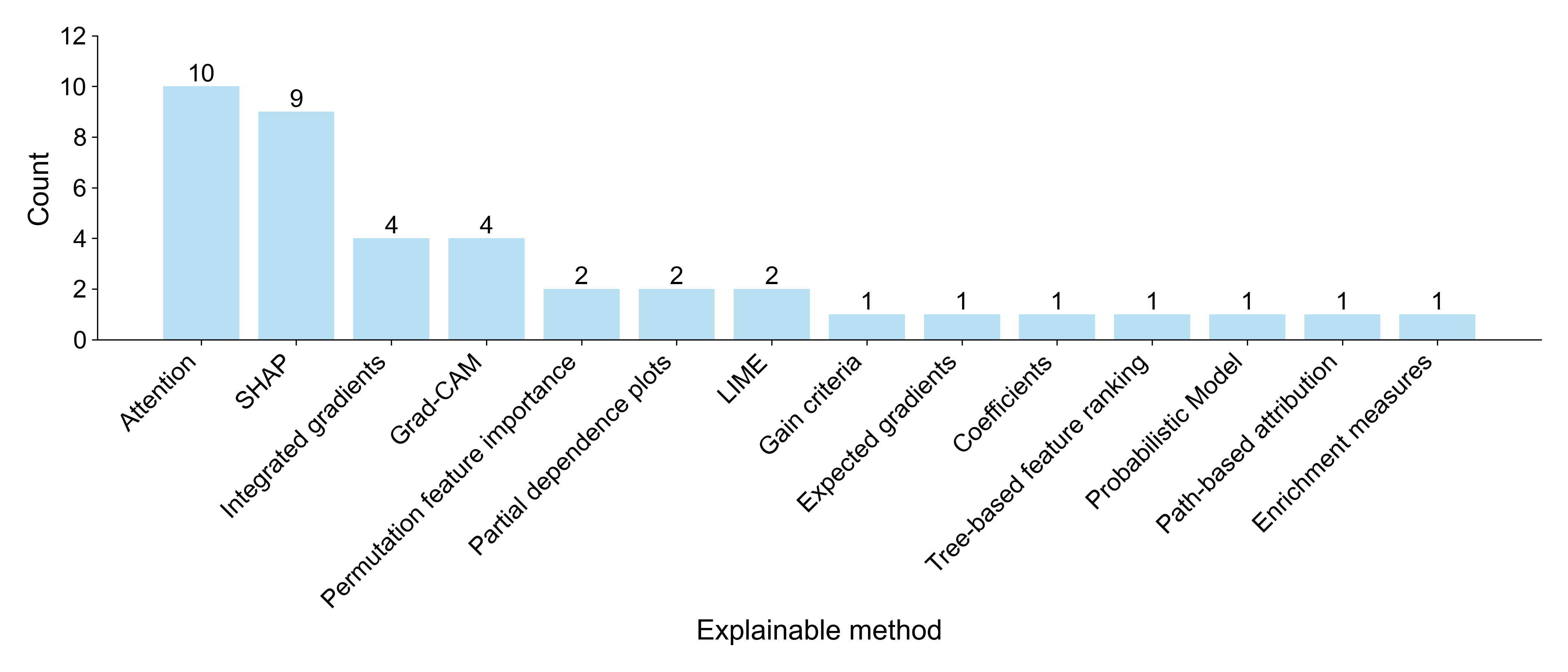


Appendix Figure 3. Explainability methods applied across 32 publications (Individual studies may employed more than one method). Grad-CAM = Gradient-weighted Class Activation Mapping, LIME = Local Interpretable Model-agnostic Explanations, SHAP = SHapley Additive exPlanations.

**References**

1. Niu, Y.*, et al.* Explainable machine learning model based on EEG, ECG, and clinical features for predicting neurological outcomes in cardiac arrest patient. *Sci Rep* **15**, 11498 (2025).

2. Kim, T.J., Suh, J., Park, S.H., Kim, Y. & Ko, S.B. System for predicting neurological outcomes following cardiac arrest based on clinical predictors using a machine learning method: the neurological outcomes after cardiac arrest method. *Neurocrit Care* **42**, 829-838 (2025).

3. Mendoza, A., Tume, S., Puri, K., Acosta, S. & Cavallaro, J.R. Clinical features and physiological signals fusion network for mechanical circulatory support need prediction in pediatric cardiac intensive care unit. *IEEE J Biomed Health Inform* **29**, 783-791 (2025).

4. Lin, M.*, et al.* An empirical study of using radiology reports and images to improve intensive care unit mortality prediction. *JAMIA Open* **8**, ooae137 (2025).

5. Agomuo, O.C., Khan, Z., Otuomasirichi, A.I., Uzoma, A.K. & Muzamal, J.H. Data-driven decision management: multi-modal analytics in a health case study. in *2025 19th International Conference on Ubiquitous Information Management and Communication* 1-6 (2025).

6. Chen, S., Wang, D., Che, C., Wei, Z. & Zhong, Z. A heterogeneous data fusion mortality prediction model based on time-aware self-attention mechanism. *Int J Mach Learn Cybern* (2025).

7. van Slobbe, R.*, et al.* Multimodal convolutional neural networks for the prediction of acute kidney injury in the intensive care. *Int J Med Inform* **196**, 105815 (2025).

8. Fang, S.*, et al.* Cross-modal similar clinical case retrieval using a modular model based on contrastive learning and k-nearest neighbor search. *Int J Med Inform* **193**, 105680 (2025).

9. Guo, J.*, et al.* Uncertainty-aware dynamic fusion for multimodal clinical prediction tasks. in *2025 IEEE International Conference on Acoustics, Speech and Signal Processing* 1-5 (2025).

10. Koyner, J.L.*, et al.* Multicenter development and validation of a multimodal deep learning model to predict moderate to severe AKI. *Clin J Am Soc Nephrol* **20**, 766-778 (2025).

11. Liu, F.*, et al.* MetaGP: A generative foundation model integrating electronic health records and multimodal imaging for addressing unmet clinical needs. *Cell Rep Med* **6**, 102056 (2025).

12. Liu, S. & Chen, H. Knowledge injected multimodal irregular EHRs model for medical prediction. in *Trustworthy Artificial Intelligence for Healthcare* 25-39 (2024).

13. Ding, S., Ye, J., Hu, X. & Zou, N. Distilling the knowledge from large-language model for health event prediction. *Sci Rep* **14**, 30675 (2024).

14. Wang, C.*, et al.* Multimodal fusion network for ICU patient outcome prediction. *Neural Netw* **180**, 106672 (2024).

15. Amiri, M.*, et al.* Multimodal prediction of 3- and 12-month outcomes in ICU patients with acute disorders of consciousness. *Neurocrit Care* **40**, 718-733 (2024).

16. Sun, M.*, et al.* A cross-modal clinical prediction system for intensive care unit patient outcome. *Knowledge-Based Systems* **283**, 111160 (2024).

17. Gao, Z.*, et al.* Improving the prognostic evaluation precision of hospital outcomes for heart failure using admission notes and clinical tabular data: multimodal deep learning model. *J Med Internet Res* **26**, e54363 (2024).

18. Lin, J.*, et al.* Development and validation of multimodal models to predict the 30-day mortality of ICU patients based on clinical parameters and chest X-rays. *J Imaging Inform Med* **37**, 1312-1322 (2024).

19. Tan, Y.*, et al.* Forecasting acute kidney injury and resource utilization in ICU patients using longitudinal, multimodal models. *J Biomed Inform* **154**, 104648 (2024).

20. Mullie, L.*, et al.* CODA: an open-source platform for federated analysis and machine learning on distributed healthcare data. *J Am Med Inform Assoc* **31**, 651-665 (2024).

21. Tiwari, N.*, et al.* MMTS: multi-modal time series based decision support system for ventilator associated pneumonia. in *2024 International Joint Conference on Neural Networks* 1-8 (2024).

22. Björkdahl, L.*, et al.* Towards holistic disease risk prediction using small language models. in *2024 International Conference on Machine Learning and Applications* 864-869 (2024).

23. Saleh, H., McCann, M., El-Sappagh, S. & Breslin, J.G. TransformerFusionNet: a real-time multimodal framework for ICU heart failure mortality prediction using big data streaming. in *2024 International Conference on Computer and Applications* 1-6 (2024).

24. Insalata, B., Schmidt, F. & Vlassov, V. Multimodal survival prediction using TabTransformer and BioClinicalBERT on MIMIC-III. in *2024 IEEE International Conference on Big Data* 1986-1992 (2024).

25. Ayden, M.A., Yuksel, M.E. & Yuksel Erdem, S.E. A two-stream deep model for automated ICD-9 code prediction in an intensive care unit. *Heliyon* **10**, e25960 (2024).

26. Cui, K., Changrong, S., Maomin, Y., Hui, Z. & Xiuxiang, L. Development of an artificial intelligence-based multimodal model for assisting in the diagnosis of necrotizing enterocolitis in newborns: a retrospective study. *Front Pediatr* **12**, 1388320 (2024).

27. Wu, Y.*, et al.* A deep learning method for predicting the COVID-19 ICU patient outcome fusing X-rays, respiratory sounds, and ICU parameters. *Expert Syst Appl* **235**, 121089 (2024).

28. Zhang, N.*, et al.* Combining biomarkers of BNIP3 L, S100B, NSE, and accessible measures to predict sepsis-associated encephalopathy: a prospective observational study. *Curr Med Res Opin* **40**, 575-582 (2024).

29. Chen, J.*, et al.* Multi-modal learning for inpatient length of stay prediction. *Comput Biol Med* **171**, 108121 (2024).

30. Ravichandran, A.M.*, et al.* XAI for better exploitation of text in medical decision support. in *Proceedings of the 23rd Workshop on Biomedical Natural Language Processing* 506-513 (Association for Computational Linguistics, Bangkok, Thailand, 2024).

31. An, Y., Qiu, R., Guo, L. & Chen, X. Attention-based multimodal fusion with adversarial network for in-hospital mortality prediction. in *2024 IEEE International Conference on Bioinformatics and Biomedicine* 1789-1795 (2024).

32. Wang, Y., Yin, C. & Zhang, P. Multimodal risk prediction with physiological signals, medical images and clinical notes. *Heliyon* **10**, e26772 (2024).

33. Zhou, Y.*, et al.* Development and validation of a deep learning-based framework for automated lung CT segmentation and acute respiratory distress syndrome prediction: a multicenter cohort study. *EClinicalMedicine* **75**, 102772 (2024).

34. Cui, S.*, et al.* Automated fusion of multimodal electronic health records for better medical predictions. in *Proceedings of the 2024 SIAM International Conference on Data Mining* 361–369 (2024).

35. Lee, K.*, et al.* Self-supervised predictive coding with multimodal fusion for patient deterioration prediction in fine-grained time resolution. in *Trustworthy Machine Learning for Healthcare* 41-50 (Springer Nature Switzerland, Cham, 2023).

36. Khader, F.*, et al.* Medical transformer for multimodal survival prediction in intensive care: integration of imaging and non-imaging data. *Sci Rep* **13**, 10666 (2023).

37. Khader, F.*, et al.* Multimodal deep learning for integrating chest radiographs and clinical parameters: a case for transformers. *Radiology* **309**, e230806 (2023).

38. King, R., Yang, T. & Mortazavi, B.J. Multimodal pretraining of medical time series and notes. in *Proceedings of Machine Learning Research*, Vol. 225 244-255 (2023).

39. Jaotombo, F., Adorni, L., Ghattas, B. & Boyer, L. Finding the best trade-off between performance and interpretability in predicting hospital length of stay using structured and unstructured data. *PLoS One* **18**, e0289795 (2023).

40. Zhang, K., Niu, K., Zhou, Y., Tai, W. & Lu, G. MedCT-BERT: Multimodal mortality prediction using medical ConvTransformer-BERT model. in *2023 IEEE 35th International Conference on Tools with Artificial Intelligence* 700-707 (2023).

41. Zhang, X., Li, S., Chen, Z., Yan, X. & Petzold, L.R. Improving medical predictions by irregular multimodal electronic health records modeling. in *Proceedings of the 40th International Conference on Machine Learning* 1731 (Honolulu, Hawaii, USA, 2023).

42. Lee, S.*, et al.* Enhancing clinical outcome predictions through auxiliary loss and sentence-level self-attention. in *2023 IEEE International Conference on Bioinformatics and Biomedicine* 1210-1217 (2023).

43. Niu, K., Zhang, K., Peng, X., Pan, Y. & Xiao, N. Deep multi-modal intermediate fusion of clinical record and time series data in mortality prediction. *Front Mol Biosci* **10**, 1136071 (2023).

44. Xu, J., Minakova, N., Sanchez, P.O. & Riezler, S. Early Prediction of Sepsis Using Time Series Forecasting. in *2023 IEEE 19th International Conference on e-Science* 1-9 (2023).

45. Kyung, J., Choi, J.H., Seong, J.S., Jeoung, Y.R. & Chang, J.H. A multi-modal teacher-student framework for improved blood pressure estimation. *Annu Int Conf IEEE Eng Med Biol Soc* **2023**, 1-5 (2023).

46. Wang, S., Liu, S. & Boutelle, M.G. Combining complementary models: fusing CNNs, RNNs, and XGBoost for enhanced outcome prediction of comatose patients after heart attack. in *2023 Computing in Cardiology*, Vol. 50 1-4 (2023).

47. Krones, F., Walker, B., Parsons, G., Lyons, T. & Mahdi, A. Multimodal deep learning approach to predicting neurological recovery from coma after cardiac arrest. in *2023 Computing in Cardiology*, Vol. 50 1-4 (2023).

48. Amiri, M.*, et al.* Multimodal prediction of residual consciousness in the intensive care unit: the CONNECT-ME study. *Brain* **146**, 50-64 (2023).

49. Lin, Y., Yu, L., Fu, Y. & Zheng, X. A predictive model for clinical health risk using multimodal electronic health record data. in *2023 the 7th International Conference on Medical and Health Informatics* 85-90 (2023).

50. Chiu, C.C.*, et al.* Integrating structured and unstructured EHR data for predicting mortality by machine learning and latent Dirichlet allocation method. *Int J Environ Res Public Health* **20**, 4340 (2023).

51. Wang, Y. & Lan, Y. Multi-view learning based on non-redundant fusion for ICU patient mortality prediction. in *ICASSP 2022 - 2022 IEEE International Conference on Acoustics, Speech and Signal Processing* 1321-1325 (2022).

52. Duvieusart, B.*, et al.* Multimodal cardiomegaly classification with image-derived digital biomarkers. in *Medical Image Understanding and Analysis* 13-27 (2022).

53. Salekin, M.S.*, et al.* Attentional generative multimodal network for neonatal postoperative pain estimation. *Med Image Comput Comput Assist Interv* **13433**, 749-759 (2022).

54. Silva, J.F. & Matos, S. Modelling patient trajectories using multimodal information. *J Biomed Inform* **134**, 104195 (2022).

55. Ahuja, Y., Zou, Y., Verma, A., Buckeridge, D. & Li, Y. MixEHR-Guided: A guided multi-modal topic modeling approach for large-scale automatic phenotyping using the electronic health record. *J Biomed Inform* **134**, 104190 (2022).

56. Jabbour, S., Fouhey, D., Kazerooni, E., Wiens, J. & Sjoding, M.W. Combining chest X-rays and electronic health record (EHR) data using machine learning to diagnose acute respiratory failure. *J Am Med Inform Assoc* **29**, 1060-1068 (2022).

57. Cheng, J.*, et al.* COVID-19 mortality prediction in the intensive care unit with deep learning based on longitudinal chest X-rays and clinical data. *Eur Radiol* **32**, 4446-4456 (2022).

58. Hayat, N., Geras, K.J. & Shamout, F.E. MedFuse: Multi-modal fusion with clinical time-series data and chest X-ray images. in *Proceedings of Machine Learning Research* Vol. 182 1-25 (2022).

59. Soenksen, L.R.*, et al.* Integrated multimodal artificial intelligence framework for healthcare applications. *NPJ Digit Med* **5**, 149 (2022).

60. Zhao, X., Wang, X., Yu, F., Shang, J. & Peng, S. UniMed: Multimodal multitask learning for medical predictions. in *2022 IEEE International Conference on Bioinformatics and Biomedicine* 1399-1404 (2022).

61. Mollura, M., Lehman, L.H., Mark, R.G. & Barbieri, R. A novel artificial intelligence based intensive care unit monitoring system: using physiological waveforms to identify sepsis. *Philos Trans A Math Phys Eng Sci* **379**, 20200252 (2021).

62. Grant, D., Papież, B.W., Parsons, G., Tarassenko, L. & Mahdi, A. Deep learning classification of cardiomegaly using combined imaging and non-imaging ICU data. in *Medical Image Understanding and Analysis* 547-558 (2021).

63. Yang, H., Kuang, L. & Xia, F. Multimodal temporal-clinical note network for mortality prediction. *J Biomed Semantics* **12**, 3 (2021).

64. Harerimana, G., Kim, J.W. & Jang, B. A deep attention model to forecast the Length Of Stay and the in-hospital mortality right on admission from ICD codes and demographic data. *J Biomed Inform* **118**, 103778 (2021).

65. Lin, M.*, et al.* An empirical study of using radiology reports and images to improve ICU-mortality prediction. in *2021 IEEE 9th International Conference on Healthcare Informatics* 497-498 (2021).

66. Shickel, B.*, et al.* Deep multi-modal transfer learning for augmented patient acuity assessment in the intelligent ICU. *Front Digit Health* **3**, 640685 (2021).

67. Salekin, M.S.*, et al.* Multimodal spatio-temporal deep learning approach for neonatal postoperative pain assessment. *Comput Biol Med* **129**, 104150 (2021).

68. An, Y., Zhang, H., Sheng, Y., Wang, J. & Chen, X. MAIN: multimodal attention-based fusion networks for diagnosis prediction. in *2021 IEEE International Conference on Bioinformatics and Biomedicine* 809-816 (2021).

69. Xu, Z., So, D.R. & Dai, A.M. MUFASA: Multimodal fusion architecture search for electronic health records. in *TheThirty-Fifth AAAI Conferenceon Artificial Intelligence* 10532-10540 (2021).

70. Yang, B. & Wu, L. How to leverage the multimodal EHR data for better medical prediction? in *Proceedings of the 2021 Conference on Empirical Methods in Natural Language Processing* 4029-4038 (Association for Computational Linguistics, Online and Punta Cana, Dominican Republic, 2021).

71. Deznabi, I., Iyyer, M. & Fiterau, M. Predicting in-hospital mortality by combining clinical notes with time-series data. in *The Joint Conference of the 59th Annual Meeting of the Association for Computational Linguistics and the 11th International Joint Conference on Natural Language Processing* 4026-4031 (Association for Computational Linguistics, Online, 2021).

72. Hayat, N., Geras, K.J. & Shamout, F.E. Towards dynamic multi-modal phenotyping using chest radiographs and physiological data. in *35th Conference on Neural Information Processing Systems* 1-5 (Sydney, Australia, 2021).

73. Bardak, B. & Tan, M. Improving clinical outcome predictions using convolution over medical entities with multimodal learning. *Artif Intell Med* **117**, 102112 (2021).

74. Hong, S.*, et al.* HOLMES: health online model ensemble serving for deep learning models in intensive care units. in *Proceedings of the 26th ACM SIGKDD International Conference on Knowledge Discovery & Data Mining* 1614-1624 (2020).

75. Darabi, S., Kachuee, M., Fazeli, S. & Sarrafzadeh, M. TAPER: time-aware patient EHR representation. *IEEE J Biomed Health Inform* **24**, 3268-3275 (2020).

76. Hammoud, I., Ramakrishnan, I.V., Henry, M. & Morley, E. Multimodal early septic shock prediction model using lasso regression with decaying response. in *2020 IEEE International Conference on Healthcare Informatics* 1-3 (2020).

77. Zhang, D., Yin, C., Zeng, J., Yuan, X. & Zhang, P. Combining structured and unstructured data for predictive models: a deep learning approach. *BMC Med Inform Decis Mak* **20**, 280 (2020).

78. Khadanga, S., Aggarwal, K., Joty, S. & Srivastava, J. Using clinical notes with time series data for ICU management. 6432-6437 (Association for Computational Linguistics, Hong Kong, China, 2019).

79. Xu, K.*, et al.* Multimodal machine learning for automated ICD coding. in *Proceedings of the 4th Machine Learning for Healthcare Conference* 197-215 (PMLR, Proceedings of Machine Learning Research, 2019).

80. Feng, Y.*, et al.* DCMN: double core memory network for patient outcome prediction with multimodal data. in *2019 IEEE International Conference on Data Mining* 200-209 (2019).

81. Jin, M.*, et al.* Improving hospital mortality prediction with medical named entities and multimodal learning. in *Conference on Neural Information Processing Systems* 1-6 (2018).

82. Xu, Y., Biswal, S., Deshpande, S.R., Maher, K.O. & Sun, J. RAIM: Recurrent attentive and intensive model of multimodal patient monitoring data. in *Proceedings of the 24th ACM SIGKDD International Conference on Knowledge Discovery & Data Mining* 2565-2573 (2018).

83. Sen, C., Hartvigsen, T., Rundensteiner, E. & Claypool, K. CREST-risk prediction for Clostridium difficile infection using multimodal data mining. in *Machine Learning and Knowledge Discovery in Databases* 52-63 (2017).

84. Huddar, V.*, et al.* Predicting complications in critical care using heterogeneous clinical data. *IEEE Access* **4**, 7988-8001 (2016).

85. Ghassemi, M.*, et al.* Unfolding physiological state: mortality modelling in intensive care units. *KDD* **2014**, 75-84 (2014).

86. Saria, S., McElvain, G., Rajani, A.K., Penn, A.A. & Koller, D.L. Combining structured and free-text data for automatic coding of patient outcomes. in *AMIA Annual Symposium Proceedings* 712-716 (2010).
